# Programming chaotic centers for shaping light branching in topological nematic vortices

**DOI:** 10.1126/sciadv.aec5012

**Published:** 2026-04-01

**Authors:** Xiao Yu, Rongxing Xu, Zuo-Xiu Tie, Ze-Yu Wang, Ling-Ling Ma, Cuiling Meng, Bing-Xiang Li, Yan-Qing Lu

**Affiliations:** ^1^ National Laboratory of Solid State Microstructures, Key Laboratory of Intelligent Optical Sensing and Manipulation, Collaborative Innovation Center of Advanced Microstructures, and College of Engineering and Applied Sciences, Nanjing University, Nanjing 210023, China.; ^2^ College of Electronic and Optical Engineering and College of Flexible Electronics (Future Technology), Nanjing University of Posts and Telecommunications, Nanjing 210023, China.; ^3^ Institute of Fundamental and Frontier Sciences, University of Electronic Science and Technology of China, Chengdu, Sichuan 610000, China.

## Abstract

Chaotic behaviors, epitomized by the butterfly effect where small causes have outsized consequences, are ubiquitous in light-matter interactions yet remain challenging to localize and even harder to engineer. Here, we demonstrate and model the direct light interacting with a programmable chaotic center—the core of photopatterning liquid-crystal topological vortices—where chaos reshapes into symmetry-protected light branching. Via confocal polarizing microscopy and Landau–de Gennes free-energy simulations, we observe the core splitting in-plane while spanning out-of-plane. This splitting pattern and peripherical director field dictate the branches number, while defect-induced refractive index variations with core-sensitive nonlinear dynamics yield distinct, spatially mapped Lorenz-like attractors. Applying a low-voltage field further allows us to reconfigure the splitting pattern and dynamically redirect the branching pathways. These findings potentially establish a versatile platform for on-chip topological photonics while serving as a laboratory analog for light scattering in extreme cosmological environments, such as near black holes.

## Teaser

Programmable topological vortices drive symmetry-protected light branching through nonlinear chaotic sensitivity.

## INTRODUCTION

The butterfly effect, a minute perturbation leading to phenomenal consequences, typifies the sensitive dependence on initial conditions that defines chaos in nonlinear dynamical systems (*1*). Such chaotic behavior, although deterministic, is inherently unpredictable and arises across a wide range of physical systems, including classical mechanical systems [e.g., the Lorenz system (*2*) and the double pendulum (*3*)], atmospheric flows (*4*), and beyond (*5*–*7*). In particular, chaotic dynamics are also widely seen in optical systems, such as nonlinear cavities and fiber networks, where instability and nonlinearity give rise to irregular emission patterns (*8*–*10*), chaotic pulse trains (*11*), and optical rogue waves (*12*, *13*). These complex behaviors, which are primarily manifested in the temporal evolution of optical fields, have been widely used in various applications, including secure optical communication (*14*), random number generation (*15*), and beam shaping (*16*), underscoring the unmet need for real-time observation (*17*).

Beyond the temporal regime, optical systems can also exhibit chaos in the spatial domain due to spatial fluctuations in refractive index. Particularly the refractive index singularities, known as optical defects, linked to the localized and strong nonlinear distortions of the material, cause light to deviate unpredictably, and make their trajectories highly sensitive to even tiny variations in initial conditions. This sensitivity forms the basis of the chaotic scattering of light, which governs light propagation in disordered systems like stadium-shaped optical resonators (*18*), turbulent fluids (*19*), or weakly random media such as soap films (*20*). When considered collectively, these scattered rays can self-organize into spatially focused, tree-like transmission channels, known as branched flows of light (*20*, *21*). This fundamental wave phenomenon, originally investigated in electronic systems, has since been identified across a variety of physical contexts, including water waves (*22*, *23*), sound waves (*24*), and optical waves (*20*). However, owing to their inherent complexity and strong dependence on correlated disordered potentials, wave branching channels have rarely been controllably shaped or integrated into singular chaotic centers exhibiting nonlinear responses (*16*, *20*, *25*, *26*). Moreover, stabilizing and harnessing controllable disordered systems—even those exhibiting chaotic points—has remained a persistent challenge, underscoring their potential for encoding higher informational entropy in secure optical communication.

**Fig. 1.**
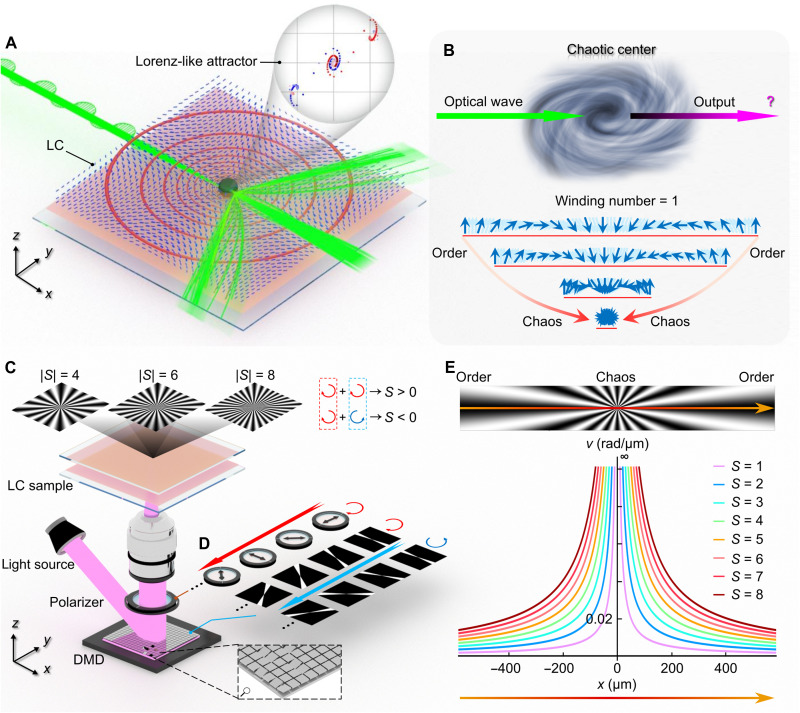
Light-chaotic center interaction and deterministic customization of topological vortices. (**A** and **B**) The main concept of this work: direct interaction of light with topological vortex cores (A) and the chaotic properties arising from drastic director rotation (B). The inset in (A) presents a key analytical result of our chaotic system, showing a Lorenz-like attractor obtained by varying the incident conditions. (**C**) Schematic of the DMD-based lithography system used for patterning LC vortices. The dashed box in the bottom-right inset shows the micromirror array of the DMD chip. Simulated microtextures of vortices with |*S*| = 4, 6, and 8 are shown in the top insets. (**D**) Multistep photopatterning process with synchronized rotation of the polarizer and uploaded subregions, controlled by a computer program. The sign of the winding number is defined by the relative rotation direction of the polarizer and subregions: positive when both rotate in the same direction. (**E**) Chaotic nature of the vortex core, revealed by the sharply increasing rotational velocity of the orientation of LC molecules. The incident beam propagates along the *x* axis.

Liquid crystals (LCs), with their self-assembled architectures and high sensitivity to external stimuli, offer an ideal platform for realizing reconfigurable disordered optical systems (*27*, *28*). Here, we demonstrate the direct light interaction with a spatially localized chaotic center in a nematic liquid crystal (NLC) system, shaping symmetry-protected optical branching with steerable channels (Fig. 1, A and B). As anisotropic fluids, NLCs are known to host a fascinating variety of topological structures (*29*, *30*). By tailoring the confining surfaces with vortical-like patterns that couple to the local director field **n**(**r**), we created complex but well-defined topological vortices, which fully reproduced the simulated patterns but are rarely found in nature. Such topological patterns are classified by the first homotopy group $\pi_{1}\left(S^{\prime }\right)=\mathbb{Z}$ and distinguished by a topological charge (also named by winding number) defined as the total rotation angle of the director field as one encircles the vortex core once, divided by 2π (Fig. 1B) (*31*). These engineered defect centers scatter light and produce controllable branched flows of light: The absolute value of topological charge controls the number of branches, while the in-plane azimuthal alignment of the director steers their propagation.

To uncover the underlying mechanism behind this behavior, we perform the simulations based on geometric optics. We find that the observed branching pattern arises as a collective outcome of rays undergoing sensitive scattering near the defect core—a hallmark of chaotic dynamics. We further clarify that the chaotic scattering of light is driven by the strong nonlinearity—analogous to the butterfly effect—originating from the strong orientational variations near the defect core and the intrinsic anisotropy of the LC medium. Within this chaotic regime, the scattered rays self-organize into multiple attractor-like trajectories that form distinct branching paths. We reveal that these branches are exactly channels where the director of the LC molecules is nearly orthogonal to the steering direction corresponding to extrema in the effective ray index. Our theoretical analysis fully foresees what we observed in the experiments, thus providing a unified framework that links topological structure and optical chaos in an emergent way.

## RESULTS

### Nematic vortices and programming chaotic centers

Topological vortices, also known as topological defects, commonly arise in NLCs when the nematic is subjected to external stimuli, such as rapidly quenching from an isotropic to a nematic phase (*32*), or electrically switching NLCs between two distinct states (*33*, *34*). The formation of topological defects, in general appeared as schlieren textures under polarized microscopy, is caused by a discontinuity in the orientational direction of the NLCs. These naturally formed defects, however, are limited in their topology diversity and spatial locations, thus posing challenges for their fundamental and functional exploration. To trans-pass this limitation, here, we used an azobenzene dye material, of which molecules show anisotropic response to the polarized purple light, to set up preferred boundary conditions and architect assorted high winding number topological defects that are rarely reported in previous works (*35*, *36*). Specifically, the orientation of rod-like azobenzene dye can template the director field of NLC molecules via the interaction of LC-dye interface, which, in turn, is prescribed by the activated light polarization (see Materials and Methods and fig. S1) (*37*, *38*). By encoding in-plane varying orientation on the azobenzene dye layer with high resolution down to submicrometer, precise director field **n**(**r**) of LC molecules can be formed consistently. In our experiments, we used a digital micromirror device (DMD)–based photopatterning system to map localized director patterns with precision ~1 μm/pixel (Fig. 1C). Using a multistep lithography approach (Fig. 1D), we precisely programmed director landscapes, successfully constructing topological vortices with winding numbers up to *S* = 8, detailed in the following section. It is worth mentioned that the assumed deformation rate of the director field (which corresponds to the optical axis) increases sharply near the vortex core (Fig. 1, B and E), giving rise to a chaotic singularity that manifests as an isotropic phase in real space. Such extreme deformation of director landscape makes the optical response highly sensitive to the initial conditions, even minute variations in the entry point of a ray can lead to markedly different trajectories. In practical experiments, the incident beam has a finite radius, naturally samples this sensitive region, which unexpectedly gives rise to Lorenz-like attractors (inset of Fig. 1A), as discussed in later sections.

To architect the predesigned topological vortices, we firstly describe its unit vector in a polar coordinate system as **n** = [cos(ϕ), sin(ϕ), 0], where the azimuthal angle, ϕ(θ) = *S*·θ + *C*, determined by the winding number *S*, initial offset angle *C*, and the polar coordinate θ in the *xy* plane (*39*, *40*). In particular, the winding number in quasi–two-dimensional systems quantifies the angular velocity of director rotation as one circumvents the defect core and is defined as S=(1/2π)∮dθ. The ϕ(θ) formalism allows us to predefine arbitrary vortex structures by specifying *S* and *C*. In the exposure process, the calculated director distribution was divided into multiple subregions, each image was uploaded sequentially to the DMD chip within fixed time interval. A polarizer was synchronously rotated before each exposure to reorient the polarization of the 405-nm illuminating light (Fig. 1D and details in Materials and Methods). The correlation between the polarizer’s rotation direction and that of the uploaded subregions defined the sign of the winding number *S*: *S* > 0 for corotating directions and *S* < 0 for opposite rotations. Through this process, complex vortex structures with tailored topological parameters (*S* and *C*) can be encoded into the LC alignment, effectively programming “chaotic centers” with controllable internal order. Yet a key question arises: Can the topological design of the director field steer the flow of light and reshape its propagation dynamics? How the chaotic nature of these vortex cores manifest itself in the way light interacts with them? Motivated by these questions, we investigate light-vortex-center interactions (Fig. 1B), which are distinct from previously reported studies (*16*, *41*, *42*).

### Light-vortex-core interactions and optical caustics

**Fig. 2.**
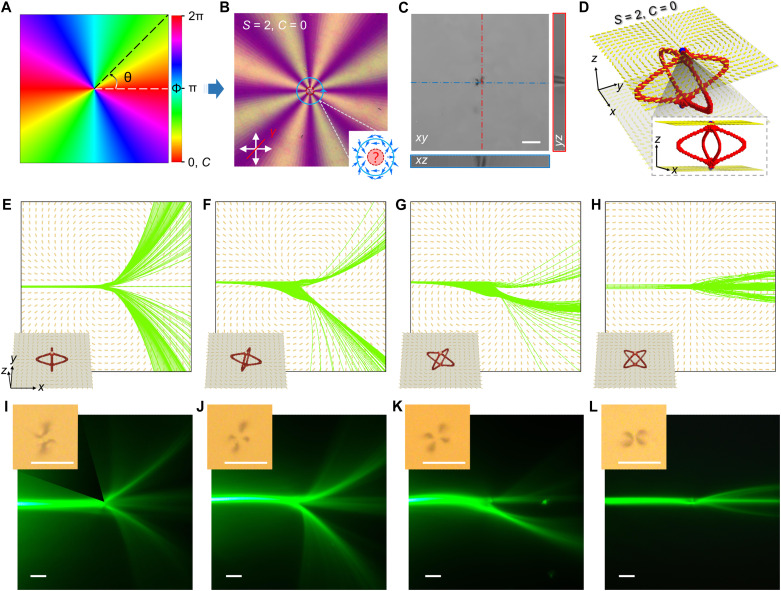
Light trajectories mediated by four distinct rotating director fields of *S* = 2 topological nematic vortices. (**A**) Schematic illustration of the designed director field in NLC, featuring vortex structures with a specified topological charge (*S*) and angular constant (*C*). (**B**) Polarizing optical micrograph of a single vortex defect with *S* = 2 and *C* = 0. The inset highlights a well-defined director configuration around the Burgers circuit (blue circle) and an undefined director orientation near the defect core (red dashed circle). Blue arrows indicate the local LC director. (**C**) FCPM images show cross-sectional views of the splitting defect cores in the central plane of the LC cell, providing both in-plane (*xy*) and vertical (*z* axis) perspectives. (**D**) Theoretically simulated expansion of defect lines (red color) emanating from splitting defect cores with *S* = 2, revealing the structure in the undefined region highlighted in (B). The yellow rods indicate the LC molecules, and the blue dots mark the defect cores on both confining surfaces. (**E** to **H**) Simulated optical trajectories after a beam encounters the defect core under various angular constants: *C* = 0 (E), π/6 (F), π/3 (G), and π/2 (H). Insets illustrate the corresponding configurations of four half-integer vortices with distinct spatial arrangements. (**I** to **L**) Experimental observations of the resulting light trajectories. Insets show bright-field micrographs of the splitting half-integer defect lines, corresponding to the simulated configurations in (E) to (H). Scale bars, 100 μm.

Topology, originally a branch of mathematics that studies properties of space preserved under continuous transformations, manifests ubiquitously across diverse physical systems—including optics (*43*), acoustics (*44*), and hydrodynamics (*45*). At the beginning, we focus on how the structural topological parameters reshape the dynamics of optical transformation. Namely, setting with *S* = 2 and *C* = 0 generates a vortex with the color-encoded azimuthal orientations as presented in Fig. 2A. The corresponding texture observed under polarized optical microscopy (POM) with a red plate compensator (λ = 530 nm) is presented in Fig. 2B, where the interference green (orange) color highlights the director aligning toward (away from) the optical axis of the compensator (*46*). The inset again depicts the director rotation in the vicinity of the core using a Burgers circuit (*39*), where the breaking of rotational symmetry of NLCs leads to an ill-defined region, behaving as the isotropic phase arrangement in the core. In particular, topological defects with the winding number of |*S*| > 1 are energetically unstable and not favored in NLC systems by splitting into half-integer counterparts (*42*, *47*). For example, the vortex with *S* = 2 spontaneously decomposes into four half-integer defects upon LC filling, forming a quatrefoil configuration as seen from the bright-field micrograph in Fig. 2C. Meanwhile, each defect core individually spans across the bulk of LC and ends at two confining surfaces as experimentally observed via fluorescence confocal polarizing microscopy (FCPM) in Fig. 2C (*48*, *49*). These isotropic defect lines, representing discontinuities of the director field, are further confirmed by numerical simulations of the scalar order parameter based on the Landau–de Gennes free-energy model, as shown in Fig. 2D. Under the combined effects of surface anchoring and free-energy minimization, the defect lines converge into point-like vertices at the substrates without inducing any director rotation out of the *xy* plane.

Notably, it is found that the pinning sites of the splitting defect lines are correlated with the initial offset angle *C*. As shown in the insets of Fig. 2 (E to H), four distinct vortices again with the same winding number of 2 but different offset angles of 0, π/6, π/3, and π/2 undergo correlative rotations of the defect lines, which collectively rotate by 0, π/12, π/6, and π/4 respectively, transitioning from a cross-shaped to a skewed, wing-like configuration. This spin behavior is also observed in the experiments [seen from the insets of Fig. 2 (I to L)] and agrees well with the numerical simulations.

To understand how this series of vortices along with rotating defect lines interplay with the light, we start with the theoretical modeling based on a Hamiltonian ray-tracing method (see Materials and Methods). This method is derived from the Fermat’s principle, which states that the light always propagates along the least optical path. Specifically, we introduced a bunch of rays (as denoted by the green lines in Fig. 2E), which precisely face the defect core and are linearly polarized along the *y* axis. As shown in Fig. 2 (E to H), the simulated light trajectories over the orientation landscapes of four vortices are vastly different. For example, vortices of *S* = 2 but with *C* = 0 and *C* = π/2 both steer the beam in a symmetrical way, where the rays are equally split into two paths toward the up- and down-side (Fig. 2, E and H). However, the splitting rays propagate differently near and after their defect cores. For instance, in the case of *C* = 0, the splitting rays become divergent as they continue to transverse away from the core, while in the case of *C* = π/2, two splitting rays intersect near the defect core and form a well-defined optical caustic, characterized by a cusp-like ray singularity, and then form three filaments with nice confinement (Fig. 2H) (*50*–*52*). For the other two vortices with intermediate offset angles, the flow of introduced rays is split into two main streams asymmetrically. Overall, as *C* increases from 0 to π/2, the incident rays undergo deflection near the defect core with two separated flows slowly reducing their deflected angles (Fig. 2, E to H) and intersecting with each other by forming three discrete branches. In experiments, we coupled a Gaussian beam with a moderate beam power, i.e., around 3 mW to ensure that the observed effects arise purely from the present director field, rather than a reoriented field induced by the nonlinear effect (*53*). It turns out, unexpectedly, that the light beams transverse through the vortices in a way that is fully consistent with the simulation (Fig. 2, I to L). An optical caustic develops once again in the experiment as vividly shown by the optical micrograph in Fig. 2L.

Unexpectedly, it turns out that the ray singularities consistently occur in the cases of *C* = π/2 across different vortices such as *S* = −2 and |*S*| = 1 systems (figs. S2 to S4), indicating a robust, symmetry-governed mechanism for caustic formation. This robustness further demonstrates that even minute variations in the ray entry position can produce sharply divergent trajectories, thereby directly linking the internal geometry of the vortex core to the observed chaotic scattering behavior. Here, we emphasize that the light trajectory can be accurately determined by the director field in the *xy* plane giving invariant **n**(**r**) along the *z* direction, while the pinning configuration of the 1/2-defect line through the *z* direction plays a distinctive role on the branching symmetry. Since the surrounding director field and the vortex core are inherently intertwined, it is difficult to disentangle their individual contributions to the beam transition dynamics. To validate the core’s influence, we fabricated three vortices with *S* = 2 and *C* = π/2 by exposing the azobenzene layer to different light energies (fig. S5). The exposing energy, yet varies, meets the minimal value to saturate the continuously varying alignment of azobenzene molecules in general and ensure a same peripheral **n**(**r**) but causes subtle differences in the splitting distance and pinning arrangement of the 1/2 motifs as shown in fig. S5B. The generated director fields, as a result, steer the light beams distinctly, where only LC vortex made by exposing proper energy (e.g., few joules per square centimeter) yields the most consistent defect arrangements and the formation of optical caustic as reliably foreseen by numerical simulations. This observation underscores the role of topological symmetry and the splitting pattern in shaping light trajectories and suggests a potential strategy for generating and tuning optical branching—a concept we explore further in the following sections.

### Branched flow enabled by topological high–winding number vortices

**Fig. 3.**
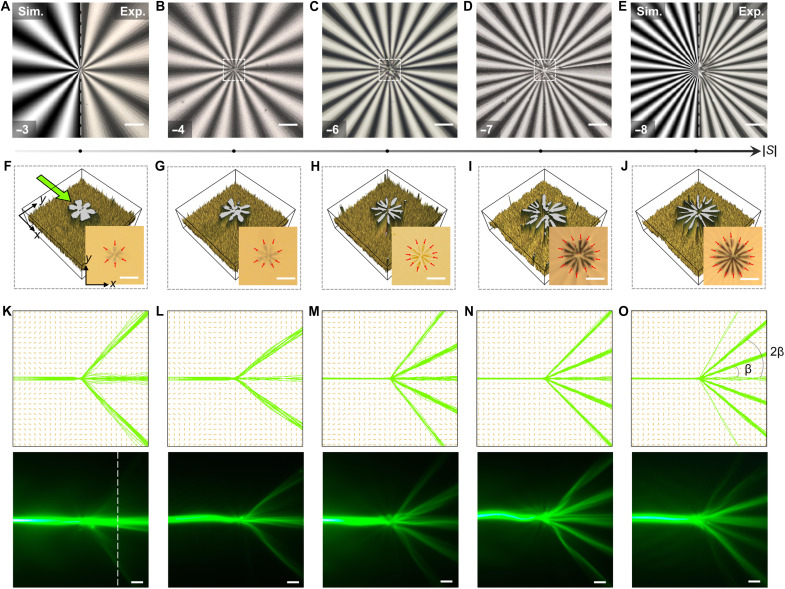
Emergent light branching triggered by vortices core with growing winding numbers. (**A** to **E**) POM images of vortex defects with specified winding numbers (as marked in the bottom-left corner) and a fixed angular constant (*C* = π/2). Simulated POM images under the same conditions are included in the left in (A) and (E). (**F** to **J**) Bright-field microscopy images (as shown in bottom-right corner) and corresponding optical intensity maps showing the splitting of defect cores under each topological condition. Red arrows indicate the number and spatial distribution of the resulting −1/2 vortices. (**K** to **O**) Simulated (top) and experimentally observed (bottom) splitting patterns of incident light field, with the number of output branches varying from 1→3 to 1→7 as the winding number increases. The inset in (O) shows the deflection angles of the branches, each separated by a uniform interval denoted as β. Scale bars, 100 μm.

Topological nematic vortices with high winding numbers even allow for more notable optical branched flows, where the optical caustic is consecutively developed. By using the high-precision photopatterning system, we successfully fabricate various high–winding number vortices, with their optical and topological characteristics well agree with the simulated ones (Fig. 3, A and E). The topological properties are inherently characterized by the optical microtextures. Namely, the vortex with *S* = −8 exhibits 32 dark brushes, with an interval of two dark brushes corresponding to a π/2 director rotation and a total direction rotation reaching 2880°. Although such large rotation angle is set up along one single loop, the experimental texture yet closely mirrors the corresponding simulated POM based on the Jones matrix method (see Materials and Methods) by matching the pixel resolution, with a calibrated scale of 1.08 μm per pixel (Fig. 3E). More essentially, despite the increasing of the winding number, the core of the vortex unambiguously decomposes into a group of *S* = −1/2 elementary ones, with the number being 2|*S*| as showed by the bright-field images in the insets of Fig. 3, F to J. Upon inverting the image’s intensity, the visibility of spatial distribution of the splitting defect lines is greatly enhanced as seen from Fig. 3 (F to H), with the splitting pattern appearing as hexagon, octagon, and dodecagon in order, reminiscent of two-dimensional crystalline packing under radial confinement. Because of the rapid increase in core splitting with winding number, high–winding number vortices require careful imaging optimization. As shown in Fig. 3 (I and J), the vortex cores with *S* = −7 and *S* = −8 were imaged using a low-magnification objective [2×, numerical aperture (NA) = 0.06] with a long depth of field (~0.15 mm), which provides a natural thick-slice projection and therefore enhances the contrast of the three-dimensional splitting structure. The increasing number and characteristic size of the split defect cores are also reproduced in the simulations as manifested in fig. S6, where both the director field and the scalar order parameter distributions are visualized.

Unlike the stochastic nature of the conventional branched flow, we demonstrate here a symmetry-protected branching behavior enabled by the isotropic-phase defect cores, in which the underlying optical potential remains fully disordered. As vividly shown by both experimental and simulated results in Fig. 3 (K to O), the optical branched flow emerges when waves propagate and meet the vortex’s core, manifesting focused filaments and tree-like caustic patterns. The ray singularities are preserved within the core regions, enabling one-to-*N* optical branching pathways, with configurations ranging from 1→3 to 1→7 as |*S*| increases from 3 to 8 (Fig. 3, K to O). Our experimental observations along with theoretical simulations of this flow of rays indicate that the optical branching here is due to iterative cusp formation of the ray paths over a variation landscape of the refractive index.

From a refractive index perspective, a topological vortex combines a disordered core with periodically aligned peripheries, enabling intensely chaotic light perturbation at the core while evenly discrete branches propagation through the refractive index channels. On one hand, the core-mediated perturbation is derived from an underlying nonlinear dynamical process, which will be discussed in details in the following section. On the other hand, the periphery-modulated steering is determined by a higher refractive index seen by the extraordinary beam, where the director **n**(**r**) is locally oriented perpendicular to the propagation direction to ensure strong confinement. Although the overall optical patterns show distinction between *C* = 0 and *C* = π/2, the high–refractive index preference consistently governs the ray trajectories regardless of the value of *C*. As shown in fig. S6D, for *C* = 0, the central propagation path corresponds to the lowest refractive index, causing the rays to diffract before reaching the core and forming a ray envelope around it. While multiple branches still emerge in the high–winding number case of *C* = 0, these separated channels develop without forming cusp-like intersections near the core region, resulting in an uneven intensity distribution (fig. S6D). As previously discussed for caustic formation in *S* = ±1 (fig. S3) and *S* = ±2 (Fig. 1 and fig. S2), the pronounced branching field observed at higher *S* again highlights that the director symmetry governed by *C* dictates the onset of optical branched flow. Therefore, we focus on the case of *C* = π/2, where the incident beam remains well collimated before reaching the vortex core, enabling remarkable topological control.

**Fig. 4.**
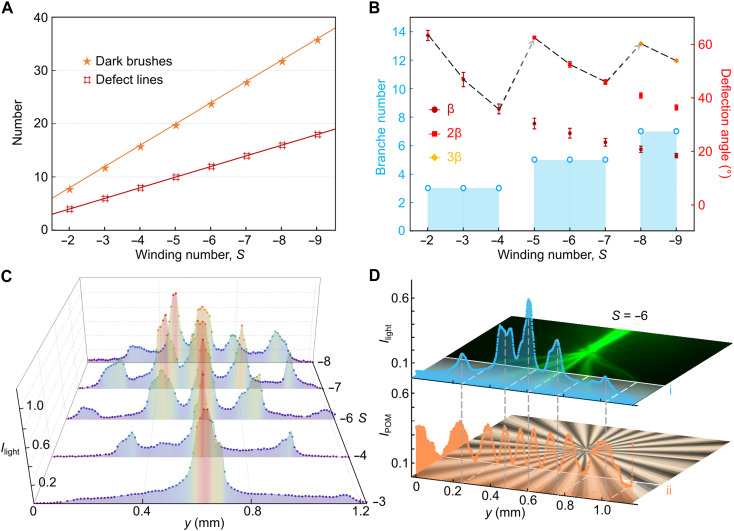
Statistical properties of evolving branching fields from chaotic centers. (**A**) Summary diagram showing the evolution of two characteristic quantities of vortices—number of dark brushes and defect lines—as a function of winding number (*S*). (**B**) Schematic illustrating variations in optical properties with winding number, including the number of branches and the deflection angles of individual branches. The dashed line indicates the trend of the outermost branch deflection angle. (**C**) Spatial transition of the branching channels along the transverse (*y* axis) direction, presented as a composite of stacked cross-sectional intensity profiles for different winding numbers. (**D**) Cross-sectional intensity profiles of the branched beams (top) and corresponding POM textures of the nematic vortices (bottom), captured at identical propagation distances (indicated by dotted lines i and ii), highlighting the spatial alignment between light branches and defect structures.

As plotted in Fig. 4 (A and B), the number of optical branches is positively correlated with the number of dark brushes or elementary defect lines. In Fig. 4B, we statistically analyze the deflection angle of each branch, defined as the angle between each outer channel and the central channel (as marked in Fig. 3O). The results reveal a systematic reduction in deflection angles, likely providing spatial allowance for the emergence of additional branches in subsequent vortices. For instance, the vortices with *S* = −6 and *S* = −7 exhibit the same number of optical branches, but the vortex of *S* = −7 notably suppresses its maximal deflection angle as compared to its counterpart of *S* = −6 (Fig. 3, M and N). The newly emerged branches exhibit a consistent deflection angle of ~60°, as highlighted by the trend marked with a dotted arrow in Fig. 4B. This behavior is further supported by tracking the dynamic evolution of the cross-sectional intensity along a fixed propagation direction (Fig. 4C), as marked in the bottom of Fig. 3K.

The observed beam transformations are strongly correlated with the periodic growth of the vortex structure as the winding number increases. Notably, the light flow after splitting is dominantly confined near or inside the dark-brush region. This is revealed in Fig. 4D, where the peaks of the light intensity profile along the white dashed line are positionally matched with the dark-brush regions beneath. The evenly spaced dark brushes also allow every two adjacent branches to propagate with an equal interval (i.e., Δβ) on their steering angles (Fig. 3O). For example, intervals of 30° and 22.5° are presented in the vortices of *S* = −6 and *S* = −7, respectively. Consequently, the deflection angles of the outer branches can be expressed as β, 2β, 3β, and so on. As the interval between adjacent channels compresses, new branches emerge once the cumulative angle (*N* + 1)β approaches ~120°, where *N* denotes the number of existing branches. This principle is also evident in vortices with increasingly positive winding numbers ranging from *S* = 4 to *S* = 7 (fig. S7). We attribute this behavior to the fact that a larger winding number introduces stronger cumulative rotation within the vortex core, which enhances the effective nonlinearity of the director field and makes the central region increasingly sensitive to small variations in initial ray positions. In addition, the programmed strong nonlinearity produces more pronounced periodic modulations of the director field, creating additional refractive index–driven attractors that guide and capture the split light flows.

Last, we confirmed that the polarization of each branched channel remains confined within the plane of the NLC layer due to the transverse nature of optical beams. This behavior is confirmed by calculating the evolution of the electric field vectors along the light rays (fig. S8). In experiments, we characterized the overall polarization distribution by launching a quasi–plane wave and analyzing the output signal using a polarization-resolved microscopy system (see Materials and Methods and fig. S8). In this state, both experimental and simulated results verify that the vortex topology effectively steers the light-branching dynamics, with the electric component of light oscillated within the layer plane. This behavior reveals the intimate coupling between light polarization, the director field, and topological structures, thereby laying the foundation for exploring externally driven branching dynamics and intrinsic optical chaos.

### Field-driven reconfigurable optical branching

LCs, with their intrinsic sensitivity to external stimuli, have enabled the engineering of complex superstructures, such as three-dimensional defect lines (*29*, *54*–*56*). To explore the tunability of these structures, we applied an alternating voltage signal at 1 kHz to NLC samples to manipulate the defect lines network and resultant optical branching behavior. The substrates were coated with transparent indium tin oxide (ITO) electrodes, enabling the establishment of a uniform electric field such as **E** = (0, 0, *E*_z_) across the gap. Owing to the positive dielectric anisotropy of the NLC material ($\Delta \varepsilon =\varepsilon_{//}-\varepsilon_{⊥}>0$), the in-plane director **n** = (*x*, *y*, 0) reorients toward the applied field direction and eventually aligns to the longitudinal direction **n** = (0, 0, 1) under sufficiently high voltage (*40*).

**Fig. 5.**
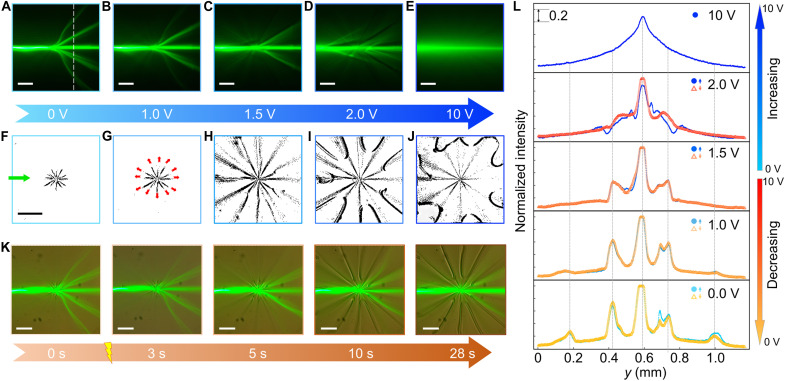
Electrically tunable light branching associated with morphed defect lines. (**A** to **E**) Representative optical trajectories corresponding to steady states under a high-frequency electric field (*f* = 1 kHz) with increasing applied voltages: *V* = 0, 1, 1.5, 2, and 10 V. The optical landscape is formed by vortex defects with topological charge *S* = −6 and *C* = π/2. (**F** to **J**) Bright-field microscopy images of defect line expansion, binarized by using the same intensity threshold. Red arrows indicate the expansion direction of each half-integer defect from the vertical to the horizontal plane under the same conditions as in (A) to (E). (**K**) Composite micrographs showing the evolution of optical trajectories and the corresponding defect fields at various relaxation times after the voltage loading (*V* = 1.5 V). Each panel simultaneously captures both the light propagation path and bright-field image of the evolving defect line structures. (**L**) Cross-sectional intensity profiles at a fixed propagating distance [marked by the dotted line inset at (A)], showing the reconfigurable beam propagation during both voltage ramp-up and ramp-down processes. Scale bars, 200 μm.

The dynamic evolution of the branched flow dependent on the applied voltage is experimentally presented in Fig. 5 (A to E) and movie S1. As the voltage increases gradually from 0 to 10 V, the optical branching pattern undergoes multiple distinct intermediate states. For example, in the vortex of *S* = −6, *C* = π/2, as the voltage increases to 1 V, the original five-branch configuration evolves into a three-branch structure, with two outermost branches dissipated (Fig. 5, B and C). This dissipation is probably due to the diminished variation on the refractive index seen by the extraordinary wave upon the application of the electric field, since there is no apparent change in the defect lines (Fig. 5, F and G). As the voltage continues to increase to 1.5 to 2.0 V, the newly formed director field can yet sustain the three-branch pattern, while this could either be ascribed to the director reorientation or, more essentially, caused by the dynamic expansion and morphological reconfiguration of the defect lines. As shown in Fig. 5 (F to I), the group of defect lines gradually morphs by collectively extending from the *z* axis to the *xy* plane and lastly adopting an in-plane configuration (Fig. 5, H and I). As the voltage becomes sufficiently high such as 10 V, only a singular path along its initial direction remains (Fig. 5, E and J), given that the director field of LC molecules is close to a uniform alignment along the *z* axis under 10 V.

To further elucidate the correlation between beam propagation and the evolution of defect lines, we recorded the dynamic response of the system under a constant applied voltage (i.e., 1.5 V) at varying field-effected durations (Fig. 5K and movie S2, S3). Using bright-field microscopy, both the optical field and the topological structure were simultaneously captured. At the beginning such as 0 to 5 s, the two outermost branches gradually disappear, and only three branches are left at the fifth second. However, the outermost optical branches reappear at the 10th second, caused by the line extension in the radial direction. The line extension lastly stabilizes into a steady-state configuration after ~28 s. This seconds-level timescale enables us to exclude molecular reorientation as the cause of the altered light trajectory, given that LC molecules realign under an applied electric field within merely tens of milliseconds. Overall, this observation confirms the dynamic coupling between defect line reconfiguration and light trajectories. In addition, this voltage-driven transition on optical branching is reversible, as revealed by experimentally tracing the branching patterns during back-and-forth voltage sweeps (Fig. 5L and fig. S9). The nearly identical intensity profiles, extracted along the white dashed line in Fig. 5A, in both directions indicate excellent reproducibility. This stability arises from the reversible reconfiguration of the defect lines, which can be dynamically morphed not only by the applied electric field but also via optical tweezers (movie S4). In summary, we have demonstrated the manipulation of topological splitting through multiple routes, including topological parameters, exposure time, and external fields, thereby achieving multidimensional control over the branching fields. Moreover, the distinct electrical response further evidences the intrinsic chaos and complexity localized at the core, a topic explored in greater depth in the following section.

### Chaos shaping branched flow of light

When light propagates through an LC medium, it interacts with the anisotropic molecular orientation and experiences birefringence, splitting into ordinary and extraordinary rays characterized by different refractive indices *n*_o_ and *n*_e_, respectively. In particular, the effective refractive index of the extraordinary ray depends on the spatial configuration of the LC director field and varies with the coordinates. This mechanism enables us to develop a geometric optics framework to numerically simulate the trajectory of light, thus helping to reveal the mechanism behind the observed branching of light in NLCs.

**Fig. 6.**
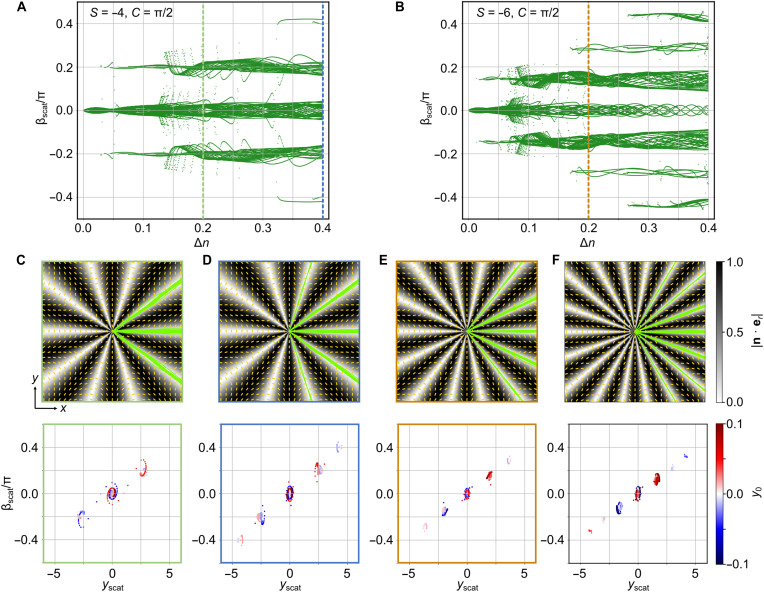
Simulations of chaos shaping the branched flow of light. (**A** and **B**) Scattering angle β_scat_ versus refractive index difference Δ*n* for defects with topological charges *S* = −4 and *S* = −6, respectively. Each green dot indicates the scattering angle of a light ray launched along the *x* axis from *y* positions within the range [−*y*_0_, *y*_0_]. (**C** to **F**) Detailed visualizations of chaotic ray scattering for different topological charges: Top shows ray trajectories (green lines) interacting with defect centers. The background color map represents the absolute value of scalar product |**n**·**e***_r_*|, where **n** is the director field and **e***_r_* is the radial vector. Bottom shows the dependence of the scattering angle β_scat_ on the transverse position *y*_scat_. The dot color encodes the initial incident position *y*_0_, and the frame color corresponds to the dashed line intervals in (A) and (B), representing the alternating conditions: *S* = −4, Δ*n* = 0.2 (C); *S* = −4, Δ*n* = 0.4 (D); and *S* = −6, Δ*n* = 0.2 (E) respectively. Subfigure (F) corresponds to the vortex with *S* = −8 and the index difference Δ*n* = 0.2. The azimuthal angles *C* of the nematic vortices discussed in this figure are all set to be π/2.

As demonstrated in Fig. 6 (A and B), rays launched with slightly different initial *y* positions but sharing the same incident direction are scattered into distinct output channels characterized by well-separated deflection angles β_scat_. This sensitive dependence on initial conditions, also experimentally demonstrated in Figs. 2 (I to L) and 3 (K to O), is a hallmark of chaotic scattering, indicating that the observed branching arises from an underlying nonlinear dynamical process. Quantitatively, light propagation in such a medium can be mapped onto the geodesic motion of a particle on a curved two-dimensional manifold, with an effective metric given by

$$g_{\mathrm{ij}}=n_{e}^{2}\delta_{\mathrm{ij}}-\Delta n^{2}n_{i}n_{j}$$ (1)

where δ*_ij_* is the Kronecker delta and *n_i_*, *n_j_* represent the Cartesian components of the director field. In this picture, the local director field and refractive indices define the geometry through which rays travel. The refractive index difference Δ*n* introduces anisotropic coupling between directions, while the spatially inhomogeneous structure of **n**(**r**), governed by |*S*|, leads to curvature in the effective metric. Together, they enhance the system’s nonlinear response. As seen in Fig. 6 (A and B), increasing Δ*n* or |*S*| results in a proliferation of well-separated output angles, signifying a transition to stronger chaotic behavior and the formation of more pronounced optical branches. This underscores the central role of topological and optical parameters in shaping the ray dynamics through enhanced nonlinearity and metric modulation.

To further investigate the spatial structure of these branches, we analyzed ray trajectories in relation to the director field **n**. As shown in Fig. 6 (C to F), the optical branches are consistently confined in the regions where the local director **n**(**r**) is perpendicular to the radial vector $e_{r}$ from the defect core, i.e., where $|n\cdot e_{r}\;|\approx 0$. This is also consistent with our experimental observation as illustrated in Fig. 4D. The **n**(**r**)-correlative confinement is not coincidental: In uniaxial anisotropic media like NLCs, light preferentially follows paths of the extrema of the effective refractive index *n*_eff_, which occurs when its propagation direction is orthogonal to **n**. Because the defect core is very small, rays scattered nearby effectively emanate from the origin, making the radial direction their postscattering trajectory. Hence, the branches emerge precisely along directions where $e_{r}⊥n$. As |*S*| increases, the azimuthal winding of **n** becomes more rapid, producing more such perpendicular alignments along the circumference. This results in a larger number of spatially localized high-index channels and thus more branches, consistent with the observed ray patterns as seen from Fig. 3 (K to O).

The fine structure of the branching is further elucidated in the bottom of Fig. 6 (C to F), which plot the scattering angle β_scat_ as a function of the initial beam position *y*_0_. These plots show that even infinitesimal differences in *y*_0_ can result in sharply different scattering outcomes, a clear manifestation of sensitivity to initial conditions, as further illustrated by supplementary experiments (movie S5). The stable output states cluster into distinct branches, forming attractor-like sets reminiscent of the “butterfly effect” in the classical Lorenz system, with deterministic sensitivity fulfilled the modern definition of Lorenz-like attractors, recently formalized through topological equivalence rather than algebraic form (*57*). These optical attractors represent the final angular states toward which rays converge after interacting with the nonlinear refractive index landscape. Their positions correspond exactly to the branches observed in the ray trajectory plots, linking the angular and spatial manifestations of chaotic scattering. It is worth noting that the Lorenz-like attractor observed in our system arises from the spatial mapping of the scattering distribution, where the scattering angle evolves as a function of the incident position and birefringence, both defined in spatial coordinates. This represents a spatial analog of the classical Lorenz attractor in a state-space projection, extending the notion of Lorenz-type chaos from temporal evolution to spatially structured optical systems.

As a result, the number and structure of branched optical trajectories in topological NLC vortices are governed by the strength of nonlinearity introduced by |*S*| and Δ*n*. The branches emerge along directions of extrema *n*_eff_, determined geometrically by the perpendicularity between the radial direction and the local director field. The scattering process is highly sensitive to initial conditions and gives rise to chaotic attractors that define the angular locations of the branches. This establishes a coherent theoretical framework linking topology, optical anisotropy, and chaotic scattering in soft photonic systems, transcending the classical understanding of topological vortices by reinterpreting them as programmable chaotic elements.

## DISCUSSION

In this work, we explored the interplay between topological defect cores and propagating light fields in NLC films, revealing a rich, chaos-mediated regime of optical branching. The topological charge and intrinsic birefringence of the medium are identified as critical parameters that drive nonlinear optical behavior, giving rise to a branched light flow that is highly sensitive to initial conditions akin to the butterfly effect in chaotic systems. Through programmable photopatterning, we precisely engineered topological singularities with tailored winding numbers and director field configurations. The internal structure of these defect cores, often hidden in traditional two-dimensional imaging, was resolved through confocal polarizing microscopy and supported by theoretical modeling. These analyses uncovered stable polygonal arrangements of split half-integer defect lines in the plane of the sample. Beyond static configurations, we demonstrated active tunability of the defect landscape. By applying external electric fields, we dynamically reconfigured defect lines from out-of-plane to in-plane orientations. This structural transition enables real-time control of the branching trajectories of transmitted light beams. Such electrically driven beam steering offers a practical route toward independent manipulation of individual optical branches—a critical functionality for the future of light-based information processing. The platform’s versatility may be further expanded by incorporating advanced LC systems such as chiral nematics or photoresponsive materials (*58*), which could offer additional degrees of topological and temporal control. Moreover, we highlight the emerging role of defect lines themselves as reconfigurable optical waveguides. Through advanced chemical doping, these defect line networks could be sculpted into sophisticated forms such as helical structures and toroidal solitons (*59*, *60*), enabling enhanced optical functionalities, thereby distinguishing such toron-forming systems from the achiral configuration explored here.

At a broader conceptual level, the observed chaotic scattering of light by topological vortex cores provides a compact laboratory analog for complex wave propagation in strongly perturbed environments—such as photon trajectories near black holes (*61*) or through turbulent interstellar media (*21*)—where extreme sensitivity to initial conditions leads to rich branching dynamics. The resulting branched flow transcends classical interpretations by combining features of chaotic response with symmetry-protected intensity profiles (*16*, *20*, *62*). This topology-controlled beam dynamics broaden the scope of topology from transformations of matter itself to light-matter interactions, establishing topology as a means for manipulating beam transitions. Enhancing the birefringence (Δ*n*) of the NLC medium—e.g., via nanoparticle doping (*63*)—may further increase the number of optical branches, especially under topological control governed by the winding number. Furthermore, recent advances in programmable LC systems have demonstrated remarkable capabilities in information display, holographic modulation, and optical encryption (*64*–*67*). Integrating our chaos-tuning mechanism with such programmable architectures could enable chaos-assisted secure optical encryption, where the sensitivity to initial optical states enhances randomness and data security. Controlled chaotic scattering may enrich dynamic display technologies by generating complex yet stable spatial textures, potentially improving viewing dimensionality, depth perception, or aesthetic diversity in future holographic and augmented-reality systems. Overall, our findings introduce a microscale platform for probing complex light-matter interactions, with far-reaching implications for soft-matter photonics, information security, and on-chip optical computing architectures.

## MATERIALS AND METHODS

### Materials and fabrication of NLC samples

To construct the NLC cell that supports in-plane light propagation, we first made an empty cell sandwiched by two ITO-coated glass substrates. The substrates were thoroughly cleaned and then treated by ultraviolet ozone for 30 min to eliminate surface contaminants and organic residues. Hereafter, the well-cleaned substrates were spin-coated with an azobenzene dye (SD1) dissolved in dimethylformamide, where the concentration of 0.7 wt % were used. The coated substrates were then baked at 100°C for 10 min to evaporate the remaining solvent. After that, two substrates were assembled into a cell using adhesive (Norland NOA-68) with the gap (30 μm) precisely controlled by silica microspheres (fig. S1). The empty cell was then transferred to a photopatterning system to set up any predesigned boundary conditions for the NLC molecules, as described in the following section. After the photopatterning, a NLC composite E7 (Jiangsu Hecheng Display Technology) was introduced into the cell by capillary force at 100°C, above its nematic-to-isotropic phase transition temperature (*T*_N–I_ = 60.5°C). At the wavelength of 532 nm and room temperature, the refractive indices of E7 for the extraordinary (ordinary) light are *n*_e_ = 1.75 and *n*_o_ = 1.52 (*68*). For FCPM measurements, the NLC E7 was doped with the fluorescent dye Nile Red (from Aldrich) at a concentration of 0.1 wt %.

### Photopatterning of topological vortices

On-demand customization of topological vortices is achieved via a photopatterning system based on a DMD device (Nanjing Ningcui Optical Technology Co. Ltd.), which leverages the intermolecular interactions between the NLC and the SD1, a polarized light-responsive material. The SD1 molecule tends to align perpendicular to the polarization of linearly polarized ultraviolet light, owing to the photo-induced trans-cis isomerization and dichroic absorption of the azobenzene moieties. The wavelength of the exposure light is selected to be 405 nm, being close to the absorption peak of the azobenzene dye (*37*). By using a rotatable polarizer, precise control over the in-plane orientation of the azobenzene dye is achieved across a 360° range in the azimuthal direction. Therefore, the pattern information is delivered to the empty cell using the DMD, which functions as a reflective spatial light modulator capable of pixel-by-pixel (1080 × 1920 pixels) deflection of incident polarized light. This anisotropic alignment of the azobenzene dye subsequently directs the orientation of LC molecules through surface anchoring interactions, forming eventual predefined director field **n**(**r**). As illustrated in fig. S1, by integrating these components into the photopatterning system, any arbitrary LC director field can be achieved with a spatial resolution of ~1.08 μm/pixel, which is much smaller than the characteristic size of the undefined region near the vortex center.

The customization of individual vortices relies on projecting the azimuthal angle of the director field into the Cartesian coordinate system, defined by the function: ϕ(*x*, *y*) = *S*·arctan(*y*/*x*) + *C*, where *S* is the winding number and *C* is the initial azimuthal offset angle. Accordingly, the local director can be expressed as **n**(*x*, *y*) = [cos(ϕ), sin(ϕ), 0]. Before patterning, the overall director distribution of the singular patterns was calculated and discretized into 72 subdomains, each assigned an azimuthal orientation ranging from 0° to 175° in increments of 5°. Consequently, each subdomain undergoes five sequential exposures, resulting in a controllable accumulated exposure dose ranging from 1 to 5 J cm^−2^. As schematically illustrated in fig. S1D, topological charges of opposite signs correspond to opposite rotational sequences of the subdomains across successive frames, while the rotation direction of the polarizer remains fixed. The rotation angle of the polarizer at each exposure step is determined by the targeted topological charge and is given by ∆ϕ = |*S*| × 2π/72. Through the multistep exposure process, the designed LC vortex pattern is imprinted onto the alignment substrates and subsequently transferred to the LC layer upon the NLC filling.

### Optical setup for beam’s incidence and detection

A continuous-wave laser beam with a wavelength of 532 nm was used to investigate the optical response of the NLC structures (fig. S8). The polarization of the incident beam was parallel to the *y* axis using a polarizing beam splitter (PBS; 50:50) and a wave plate with a half-wavelength (λ/2) retardance (JCOPTIX, China). The PBS directed the beam along the transmission path for interaction with the LC sample, while the reflected path was monitored by an optical power meter to measure the incident beam intensity. A neutral density filter was used to attenuate the laser power, ensuring a coupling power of ~3 mW at the coupling entrance. The collimated beam was then focused into the LC cell using an objective lens (Nikon, 10×, 0.5 NA). This optical configuration enabled efficient beam coupling with a waist diameter of ~15 μm, which is smaller than the thickness of the LC layer. The beam waist was precisely positioned at the cell edge using a motorized translation stage. Optical beam trajectories were captured on the basis of out-of-plane scattering, as viewed from a top-down perspective. The emergent light was collected using another objective lens and recorded with a camera of the same specifications.

### Microscopic imaging and video recording

Image and video acquisition was performed by using a high-resolution complementary metal-oxide semiconductor camera (EXMOR, SONY, Japan) with a resolution of 3000 × 3008 pixels (3.76 × 3.76 μm/pixel), combined with 2×, 5×, and 10× Nikon objective lenses with NAs of 0.06, 0.13, and 0.30, respectively. A light-emitting diode light source with a color temperature of 4000 K was used for sample illumination. The dark brushes associated with topological defects were observed under POM in crossed-polarizer mode (with a polarizer and an analyzer), enabled by the intrinsic birefringence of the NLC material. The microscope was further equipped with a full-wavelength (λ = 530 nm) compensator (commonly referred to as a “red plate”), which introduces a red background and produces distinct color contrasts between director orientations of +45° and −45°, enhancing the visualization of director configurations. Vortex lines were observed in bright-field mode without the use of the analyzer. Optical beam trajectories were captured on the basis of out-of-plane scattering, as viewed from a top and down perspective.

The topological properties of a vortex, including the winding number *S* and angular offset *C*, were characterized using POM. The magnitude of the winding number |*S*| is proportional to one-fourth the number of dark brushes. The sign of *S* was again confirmed by rotating the analyzer under crossed-polarizer conditions: if the rotation direction of the texture matches that of the analyzer, the sign is positive (movie S6). The angular offset *C* was further characterized using the red plate, which introduces color contrast corresponding to specific director orientations, thereby allowing unambiguous identification of the local director configuration.

### Statistical analysis

#### Simulation of the POM micrographs

Simulated POM micrographs were generated by calculating the transmitted light intensity using the Jones matrix method, which models the propagation of light through a polarizer, a NLC layer, and an analyzer. The simulation begins by numerically constructing the planar director distribution ϕ(x,y), which defines the local azimuthal orientation of the NLC director. When linearly polarized light passes through a birefringent medium with a spatially varying optic axis and exits through an analyzer oriented orthogonally to the input polarization, the transmitted intensity I(x,y) at each point is given by (*40*)

$$I\left(x,y\right)={\text{sin}}^{2}\left[2\phi \left(x,y\right)\right]\cdot {\text{sin}}^{2}\left(\frac{\pi }{\lambda }\Delta n\cdot d\right)$$ (2)

Here, λ is the wavelength of the incident light, ∆*n* is the birefringence of the NLC, and *d* is the thickness of LC layer. This expression is derived from the Jones matrix representation of a birefringent wave plate. The Jones matrix for a uniaxial birefringent medium with an optic axis at angle ϕ is expressed as

$$J\left(\phi \right)=R\left(\phi \right)\left(\begin{array}{cc} e^{-i\Gamma /2} & 0\\ 0 & e^{i\Gamma /2} \end{array}\right)R\left(-\phi \right)$$ (3)

where *R*(ϕ) is the rotation matrix and the phase retardation Γ is defined as

$$\Gamma =\frac{2\pi }{\lambda }\mathrm{\Delta n}\cdot d$$ (4)

When the NLC sample is placed between crossed polarizers (oriented at 0° and 90°), the transmitted intensity simplifies to the expression above, enabling the simulation of POM images corresponding to the underlying director field distribution.

#### Modeling of director field in NLCs

We numerically investigate the local director field in NLCs confined between two predesigned patterned surfaces and the ensuing defect splitting. The simulations are based on the minimization of the Landau–de Gennes free energy functional

$$F=\int_{V}f\;dV$$ (5)

where the free-energy density f is formulated in terms of the continuum tensorial order parameter **Q**

$$Q_{\mathrm{ij}}=Q_{0}\left(n_{i}n_{j}-\frac{1}{3}\delta_{\mathrm{ij}}\right)$$ (6)

Here, $Q_{0}\in \left[-1/2,1\right]$ is the uniaxial scalar order parameter characterizing the degree of local orientational order, *n_i_* are the Cartesian components (*i* = *x*, *y*, and *z*) of the director field **n**(**r**), and δ*_ij_* denotes the Kronecker delta.

Under the one-constant approximation, the free-energy density is expressed as

$$f\left(Q,\nabla Q\right)=\frac{A}{2}Q_{\mathrm{ij}}Q_{\mathrm{ji}}+\frac{B}{3}Q_{\mathrm{ij}}Q_{\mathrm{jk}}Q_{\mathrm{ki}}+\frac{C_{1}}{4}{\left(Q_{\mathrm{ij}}Q_{\mathrm{ji}}\right)}^{2}+\frac{L}{2}{\left(\partial_{k}Q_{\mathrm{ij}}\right)}^{2}$$ (7)

where *A*, *B*, and *C*_1_ are thermotropic material constants, and *L* is the elastic constant. The term $\partial_{k}Q_{\mathrm{ij}}$ denotes the partial derivative of *Q_ij_* with respect to the *k*-th spatial coordinate. The first three terms describe the thermotropic contribution driving the nematic-isotropic phase transition, while the last term accounts for the elastic distortion energy associated with spatial derivatives of the tensorial order parameter. Throughout this work, we adopt Einstein summation convention for repeated indices, consistent with standard tensor calculus as presented in general relativity textbooks (*69*).

In our simulations, Dirichlet boundary conditions, representing infinitely strong surface anchoring, are imposed on both confining surfaces, which encode an identical two-dimensional vortex pattern. The computational domain is discretized on a 128 by 128 by 32 uniform cubic grid with the grid spacing ~1 μm. The parameters are chosen according to the literature (*70*) and experimental observations: *L* = 4 × 10^−11^ N, *A* = −0.172 × 10^6^ J/m^3^, *B* = −2.12 × 10^6^ J/m^3^, and *C*_1_ = 1.73 × 10^6^ J/m^3^. The minimization of the total free energy is based on the standard procedure of calculus of variations, yielding the Euler-Lagrange equation. Then, the time evolution of the **Q**-tensor follows the Euler-Lagrange equation with a relaxation term

$$-\gamma \frac{\text{∂}Q_{\mathrm{ij}}}{\text{∂}t}=\frac{\delta f}{\delta Q_{\mathrm{ij}}}$$ (8)

where γ is the numerical relaxation constant.

With the above setups, the **Q**-tensor is numerically updated by a pseudo-gradient descent method per time interval Δ*t*, of which the pseudo-gradient is defined as the functional derivatives $\delta f\;/\delta Q_{\mathrm{ij}}$, saying

$$Q_{\mathrm{ij}}^{\text{new}}\leftarrow Q_{\mathrm{ij}}^{\text{old}}-\frac{\Delta t}{\gamma }\frac{\delta f}{\delta Q_{\mathrm{ij}}}$$ (9)

The required spatial variations of the tensorial order parameter are computed using a second-order finite-difference scheme. Upon convergence of the free energy, the local scalar order parameter *Q*_0_ is precisely 1.5 times the largest eigenvalue of the tensor **Q**, with the corresponding eigenvector defining the local director field **n**(**r**). Throughout this paper, defect lines are visualized by plotting isosurfaces corresponding to the lowest 2 to 5% of the scalar order parameter *Q*_0_.

Our numerical results reproduce experimentally observed microscopic images of vortices and the corresponding splitting defect lines in the presence of surface boundary conditions with the photo-programmable patterns.

#### Modeling of ray trajectories

We model ray trajectories in NLCs based on the framework of geometrical optics (*59*, *71*). By analogy with the principle of least action in classical mechanics, the ray trajectory is governed by the principle of least optical path, known as the Fermat’s principle. The optical path functional $S_{\text{opt}}$ is given by

$$S_{\text{opt}}=\int n_{\text{eff}}\left(s\right)\;\mathrm{ds}$$ (10)

where *n*_eff_ denotes the effective ray index associated with the direction of the energy flux along the path *s*, expressed as

$$n_{\text{eff}}=\sqrt{n_{o}^{2}{\text{cos}}^{2}\zeta +n_{e}^{2}{\text{sin}}^{2}\zeta }$$ (11)

Here, *n*_o_ and *n*_e_ represent the ordinary and extraordinary refractive indices of the nematic LC, respectively, and ζ is the angle between the optical axis (i.e., the local director field) and the direction of energy propagation (i.e., the tangent vector of the ray path).

It is noteworthy that, within a birefringent medium, a ray trajectory in Euclidean space can be equivalently interpreted as a geodesic in an effective geometry. Drawing upon classical mechanics and using the Cauchy-Schwarz inequality, we define the Lagrangian of the extraordinary ray as

$$L\left(r,\dot{r}\right)\equiv \frac{1}{2}g_{\mathrm{ij}}{\dot{r}}^{i}{\dot{r}}^{j}$$ (12)

where **r** denotes the spatial coordinates and $\dot{r}=dr/\mathrm{ds}$ is the derivative with respect to the arc length. The metric tensor *g_ij_* encodes the effective geometry and is determined by the spatial distributions of the director field

$$g_{\mathrm{ij}}\equiv n_{e}^{2}\delta_{\mathrm{ij}}-\Delta n^{2}n_{i}n_{j}$$ (13)

where the notation $\Delta n^{2}=n_{e}^{2}-n_{o}^{2}$.

Following standard procedures in field theory, the Hamiltonian is obtained via the Legendre transformation

$$H\left(r,p\right)=p\cdot \dot{r}-L=\frac{1}{2}g^{\mathrm{ij}}p_{i}p_{j}$$ (14)

where the generalized momentum is defined as $p_{i}=g_{\mathrm{ij}}{\dot{r}}^{j}$ and $g^{\mathrm{ij}}$ is the inverse of the metric $g_{\mathrm{ij}}$. Physically, the vector **p** corresponds to the normalized local wave vector, which originates from the eikonal equation in the theory of birefringence, described elsewhere (*72*).

The resulting ray trajectory is governed by the Hamiltonian system

$$\dot{r}=\frac{\text{∂}H}{\text{∂}p},\dot{p}=-\frac{\text{∂}H}{\text{∂}r}$$ (15)

Given a prescribed director field **n**(**r**), we numerically solve the above system using a fourth-order Runge-Kutta method, thus obtaining complete light trajectories through the three-dimensional topological vortex field of LC.

## Supplementary Materials

### The PDF file includes:

Supplementary Text Figs. S1 to S10 Legends for movies S1 to S6 References
