# Supplementary material for: Programming chaotic centers for shaping light branching in topological nematic vortices
Source: Sci Adv. 2026 Apr 1;12(14):eaec5012. doi: 10.1126/sciadv.aec5012 (PMC13041760; doi:10.1126/sciadv.aec5012)
Supplement: Supplementary file 1 — Supplementary Text Figs. S1 to S10 Legends for movies S1 to S6 References [file sciadv.aec5012_sm.pdf]

Science Advances
AAAS

Supplementary Materials for

Programming chaotic centers for shaping light branching in topological nematic vortices

Xiao Yu *et al.*

Corresponding author: Ling-Ling Ma, malingling@nju.edu.cn; Cuiling Meng, cuilingmeng@uestc.edu.cn; Bing-Xiang Li, bxli@njupt.edu.cn; Yan-Qing Lu, yqlu@nju.edu.cn

Sci. Adv. **12**, eaec5012 (2026)
DOI: 10.1126/sciadv.aec5012

The PDF file includes:

Supplementary Text
Figs. S1 to S10
Legends for movies S1 to S6
References

Other Supplementary Material for this manuscript includes the following:

Movies S1 to S6

Supplementary Texts

1. Reconfigurable expansion of defect lines

The spontaneous splitting of defect cores into multiple half-integer defects naturally occurs at high topological charges (S > 1), driven by the minimization of elastic free energy. The separation distance between the resulting half-integer vortices can be precisely tuned by adjusting the total radiant exposure power P, which depends on both the illumination intensity and exposure duration. In our experiments, the radiant power was controlled by alternating the exposure time of each sub-regions from 5 s to 30 s, as illustrated in fig. S5.

The in-plane expansion of defect lines from their initial vertical orientation is achieved through the application of an alternative current (AC) electric field. The ITO-coated substrates of the NLC cell exhibit a sheet resistance of 5 ohms/cm^2, enable vertical transmission of the electric field ($\mathbf{E} = (0, 0, E_z)$). The AC electric field (f = 1 kHz) was generated using a waveform generator (33522B, Keysight Technologies Inc.) and amplified via a high-voltage amplifier (ATA-2081, Aigtek).

2. Laser tweezers

A laser tweezer system was employed to manipulate the vortex cores (*73*), utilizing a semiconductor laser with a wavelength of 1064 nm (Changchun Laser Optoelectronics Technology Co., Ltd.). The laser output was continuously controlled by a power modulation unit, with a maximum output power of 2 W. To enhance experimental flexibility, the laser tweezer optical path was integrated with both the polarized optical microscope and the beam coupling setup, as illustrated in Supplementary fig. S10. The laser beam was directed perpendicularly onto the LC cell (top-down incidence) by a series of protected silver mirrors (JOPTIX, China), ensuring precise spatial alignment with the region of interest. A short-pass filter was placed in front of the camera to block scattered infrared light from the 1064-nm laser, thereby preventing imaging interference and ensuring high-contrast visualization. At an output laser power of approximately 1 W, a 10× objective lens with a numerical aperture of 0.3 was used to focus the laser beam for localized heating at the LC membrane, inducing a phase transition of the NLC from the nematic to the isotropic phase. This localized melting spot enabled the removal of micro-scale dust particles trapped within the defect core region and facilitated the shearing of connections between split vortex lines generated during the photopatterning process (Supplementary movie S4).

3. Fluorescence confocal polarizing microscopy

The morphology of isotropic vortex lines was reconstructed using both polarized optical microscopy and fluorescence confocal polarizing microscopy (FCPM) cross-sectional imaging. For FCPM measurements, the samples were fabricated using two coverslips (each 0.13 mm thick) to accommodate the limited working distance of the confocal microscope (FV3000, Olympus). A 561-nm laser was selected as the excitation source, closely matching the excitation peak of the Nile Red dye (543 nm). The confocal cross-sectional images obtained under linearly polarized excitation reflect the local director orientation, owing to the anisotropic shape of Nile Red molecules, which spontaneously align along the local nematic director **n**(**r**) (*48*). In this condition, vortex lines oriented along the vertical (out-of-plane) direction appear dark in fluorescence images due to their isotropic nature. Additionally, the configuration of the vortex lines can be reconstructed using conventional lamp illumination and undoped LC cells, providing complementary information without relying on fluorescence contrast, as illustrated in Fig. 1C.

Supplementary Figure S1

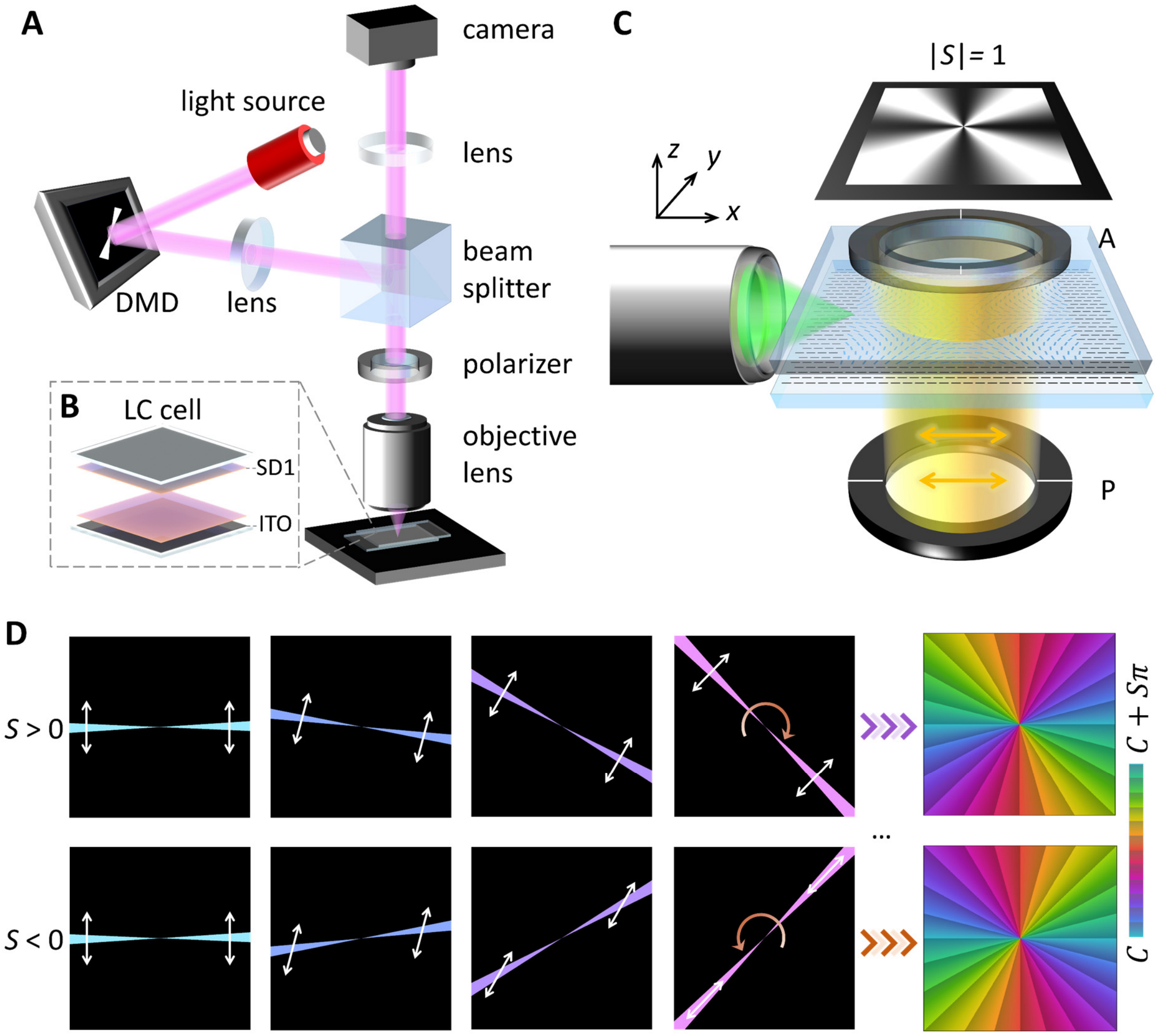

Fig. S1. Experimental setup for customization and observation of LC topological vortices. (**A**) Optical setup of the DMD-based photoalignment technique. The polarization and the intensity distribution of the exposure light are controlled by the electronic controlled polarizer and DMD chip. (**B**) Schematic diagram illustrates the construction of an LC sample, including two glasses substrates, ITO layers and alignment layers (SD1). (**C**) Experimental setup for observing in-plane beam transformation and the LC textures. The schlieren textures are obtained under cross polarizers (P and A). (**D**) Multistep exposure strategy for encoding specified intensity and polarization patterns. The bright-pixels areas denote regions of high light intensity; white double arrows indicate polarization directions. Circular arrows represent rotation direction of sub-regions used to generate vortices with different winding numbers. The color-coded diagram illustrates the final director distribution of the vortex determined by the initial director orientation (*C*), and topological charges (*S*).

Supplementary Figure S2

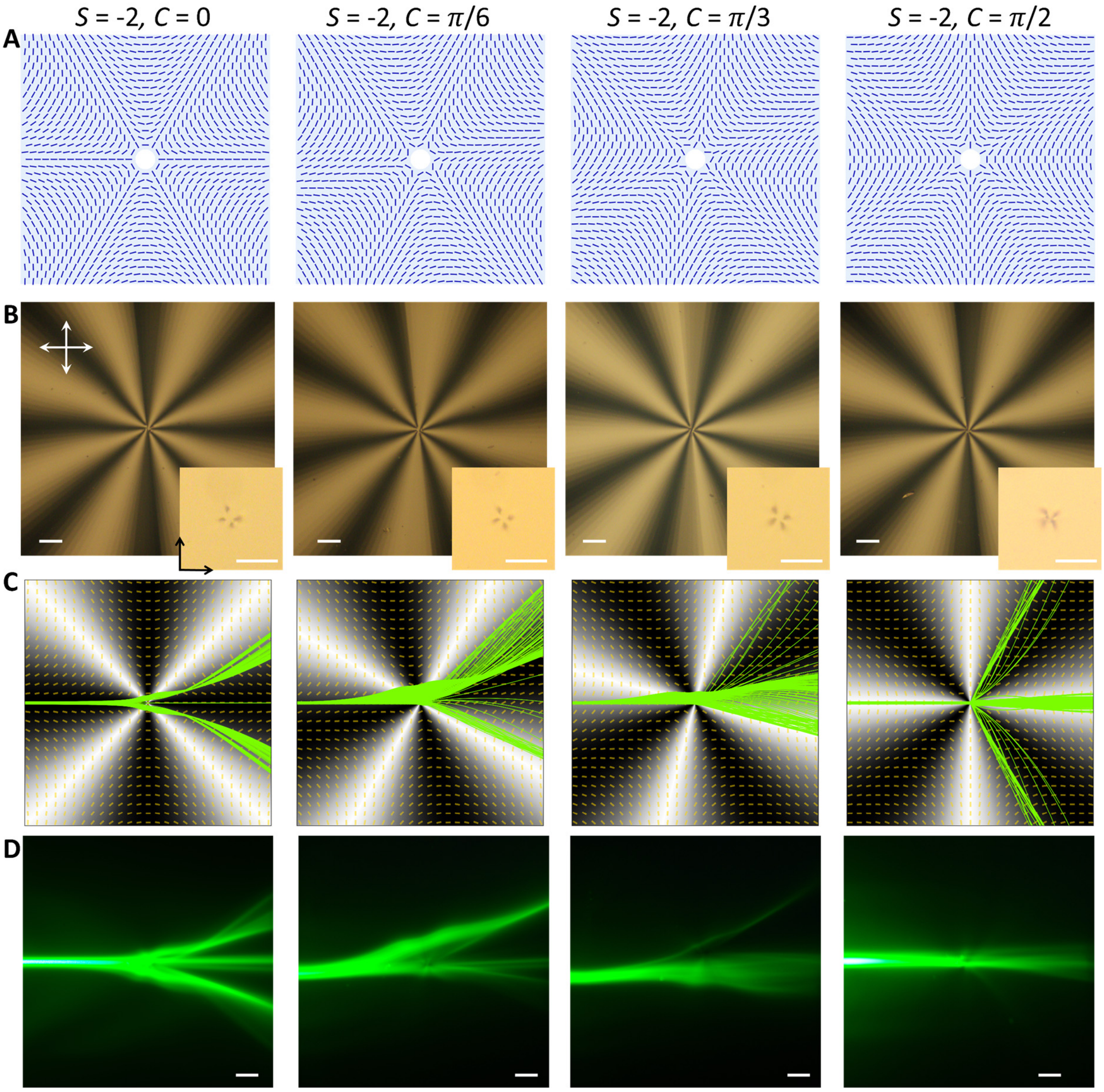

Fig. S2. light trajectories mediated by four distinct rotating director fields of *S* = -2 topological nematic vortices. (**A**) Schematic illustrations of the designed director field in NLC, featuring vortex structures with a specified topological charge (S = -2) and diverse angular constants (C = 0, π/6, π/3, π/2). (**B**) Polarizing optical micrographs of corresponding vortex defects. Inset represents the bright-field images of the defect cores illustrating the corresponding configurations of four half-integer (+1/2) vortices with distinct spatial arrangements. (**C**) Simulated optical trajectories after a beam encounters the defect field under various angular constants. (**D**) Experimental observations of the scattered optical fields under matching conditions. The scale bar represents 100 μm.

Supplementary Figure S3

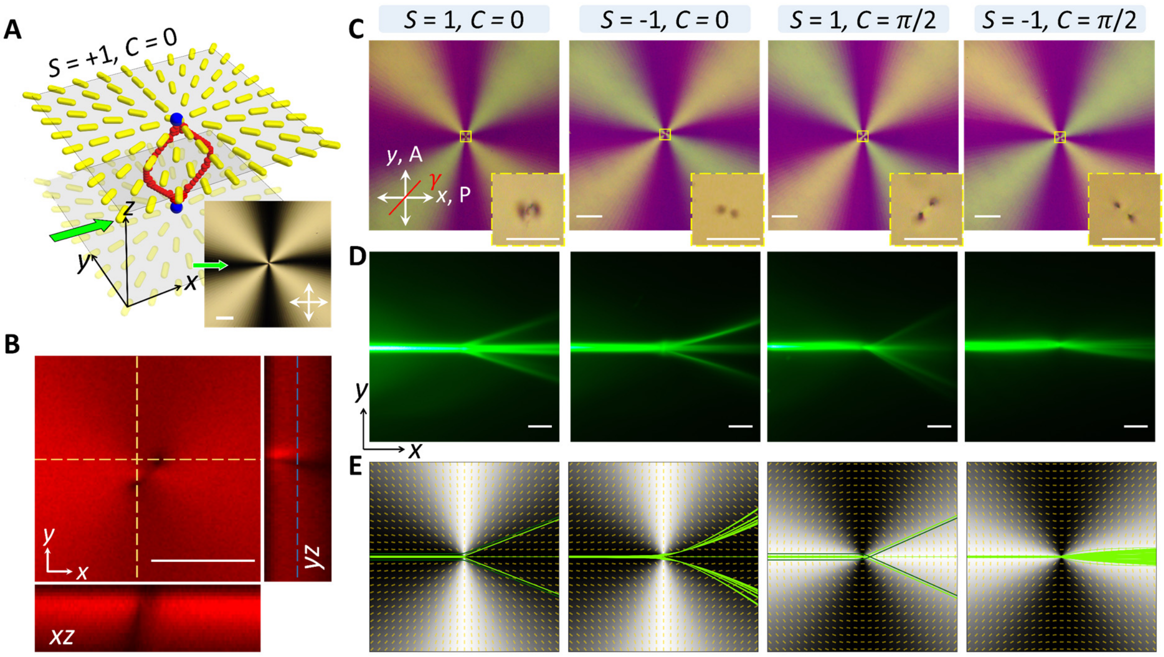

Fig. S3. Diverse optical trajectories induced by topological vortices with |*S*| = 1. (**A**) Three-dimensional schematic illustrates the splitting of defect cores for a vortex with S = 1and C = 0. The inset shows the corresponding POM micrograph. (**B**) FCPM cross-sectional image revealing the internal structure of the vortex line. (**C**) POM images of topological vortices with identical topological charge magnitude (|S| = 1) but different angular offsets ($C = 0$, π/2), observed under crossed polarizers with the addition of a full-wave retardation plate (red plate). (**D**) Micrographs of optical trajectories demonstrate distinct scattering behaviors induced by the vortex cores shown in (**C**). (**E**) Simulated light trajectories overlaid on the corresponding effective refractive index distributions. Scale bar: 150 μm.

Supplementary Figure S4

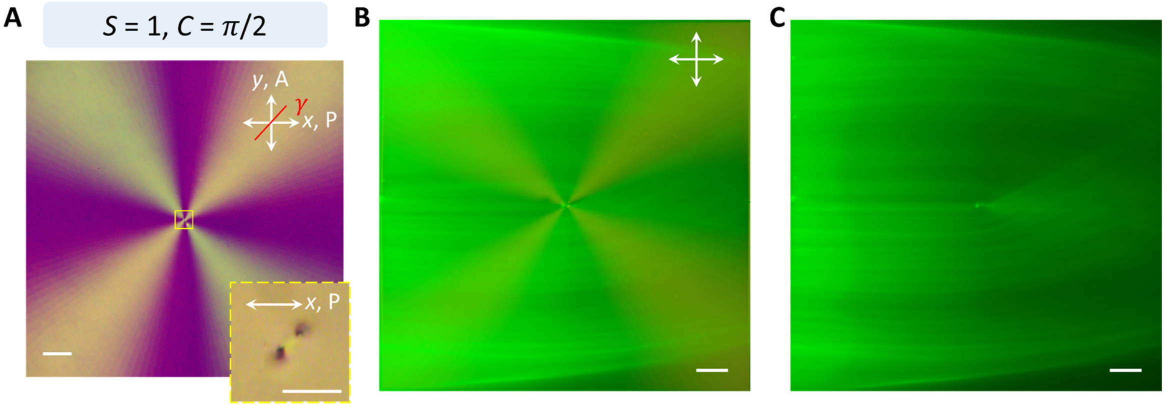

Fig. S4. Emergent optical caustics induced by topological vortices with $S = 1$, $C = \pi/2$. (**A**) POM micrographs of topological vortices with $S = 1$, $C = \pi/2$, observed under cross polarizers with the red plate. The inset diagram shows bright-field image highlighting the defect lines near the core region. (**B**) to (**C**) Optical micrographs showing the emergence of optical caustics under quasi-plane wave incidence, recorded with (B) and without (C) two cross polarizers. Scale bar: 100 μm.

Supplementary Figure S5

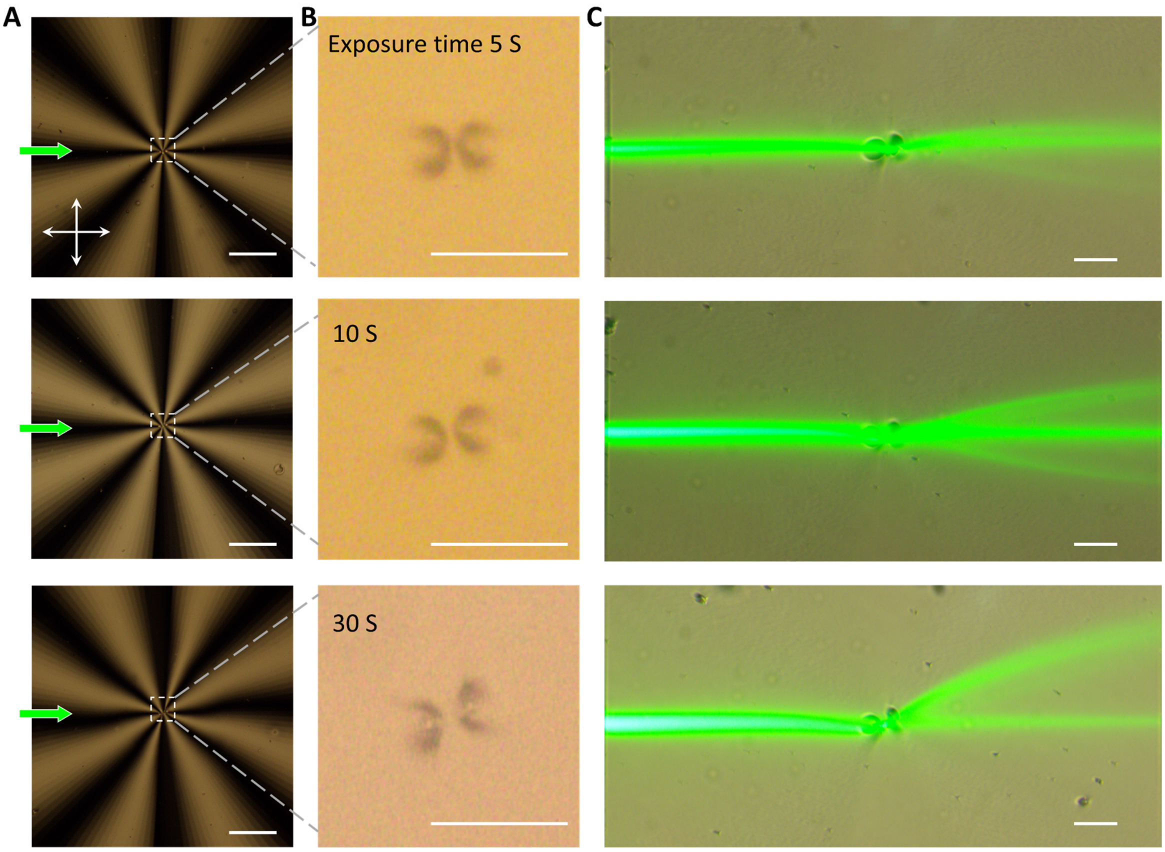

Fig. S5. Beam trajectories induced by *S* = 2, *C* = π/2 vortices with varying exposure durations. (**A**) POM micrographs showing the microtextures of topological vortices generated under different UV exposure durations for each sub-regions: 5 s, 10 s, and 30 s (from top to bottom). Even the shortest exposure of 5 s delivers an energy dose exceeding 1 J·cm^{-2}, fully sufficient to induce ordering of the azobenzene alignment layer (*74*). (**B**) Bright-field images reveal the configurations of the defect cores under corresponding exposure conditions. (**C**) Optical beam trajectories induced by the *S* = 2 vortices exhibit distinct patterns despite sharing the same topological charge, reflecting the impact of exposure time on defect morphology. Scale bar: 100 μm.

Supplementary Figure S6

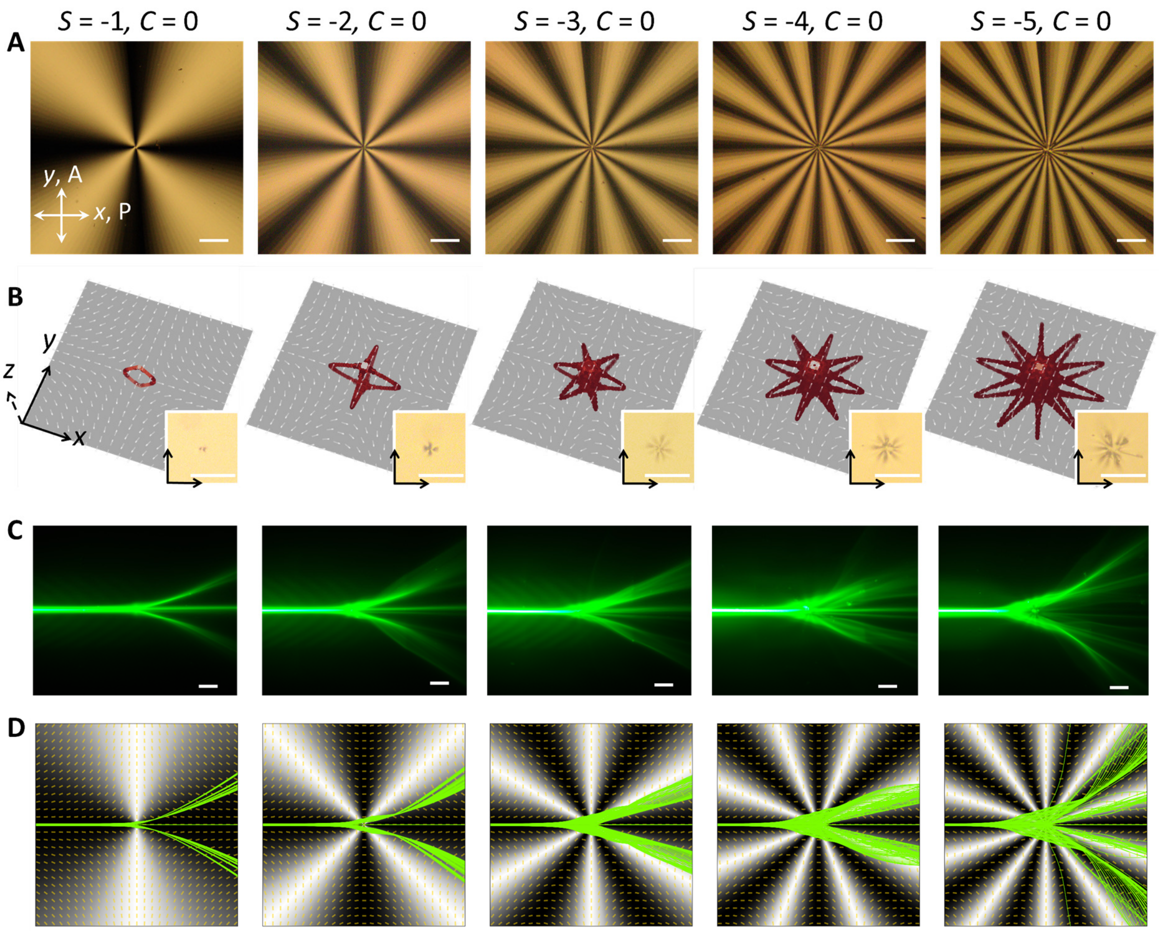

Fig. S6. Optical splitting induced by topological vortices with *C* = 0. (**A**) POM images of the topological vortices with a constant angular offset $C = 0$ displaying diverse winding numbers ranging from $S = -1$ to $S = -5$. (**B**) Numerical simulations illustrating the splitting of defect cores into multiple half-integer defects. Red dots indicate regions of reduced scalar order parameter. Insets show corresponding experimental observations of the defect splitting. (**C**) Experimentally captured light trajectories resulting from in-plane scattering through the vortex configurations shown in (A), highlighting distinct branching behaviors. (**D**) Simulated light trajectories overlaid on the background of the effective refractive index landscape. Yellow rods represent the local director distribution, emphasizing the guidance effect of the topological textures. Scale bar: 150 μm.

Supplementary Figure S7

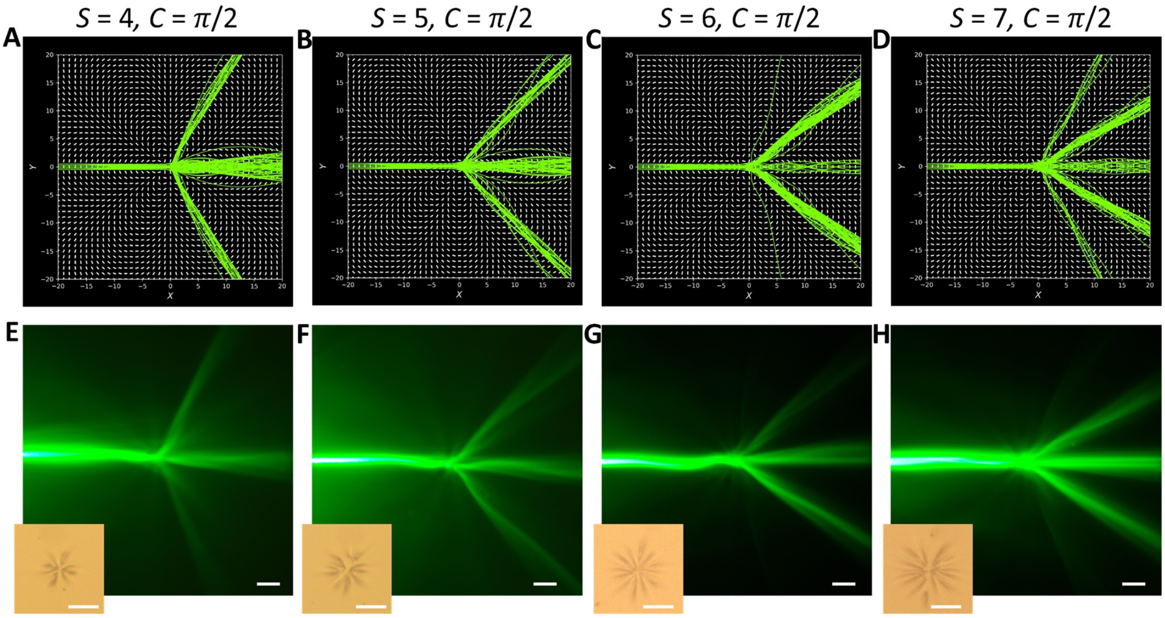

Fig. S7. Emergence of optical branching induced by topological vortices with increasingly positive winding numbers (S > 0). (**A**) to (**D**) Numerical simulations of optical branching patterns generated by topological vortices with a constant angular offset $C = \pi/2$ and winding numbers increasing from $S = 4$ (A) to $S = 7$ (D). (**E**) to (**H**) Corresponding experimental observations of the light trajectories under identical conditions. The number of optical branches increases nonlinearly with the winding number, indicating a discontinuous branching transition. Insets show bright-field micrographs of the associated defect lines. Scale bar: 100 µm.

Supplementary Figure S8

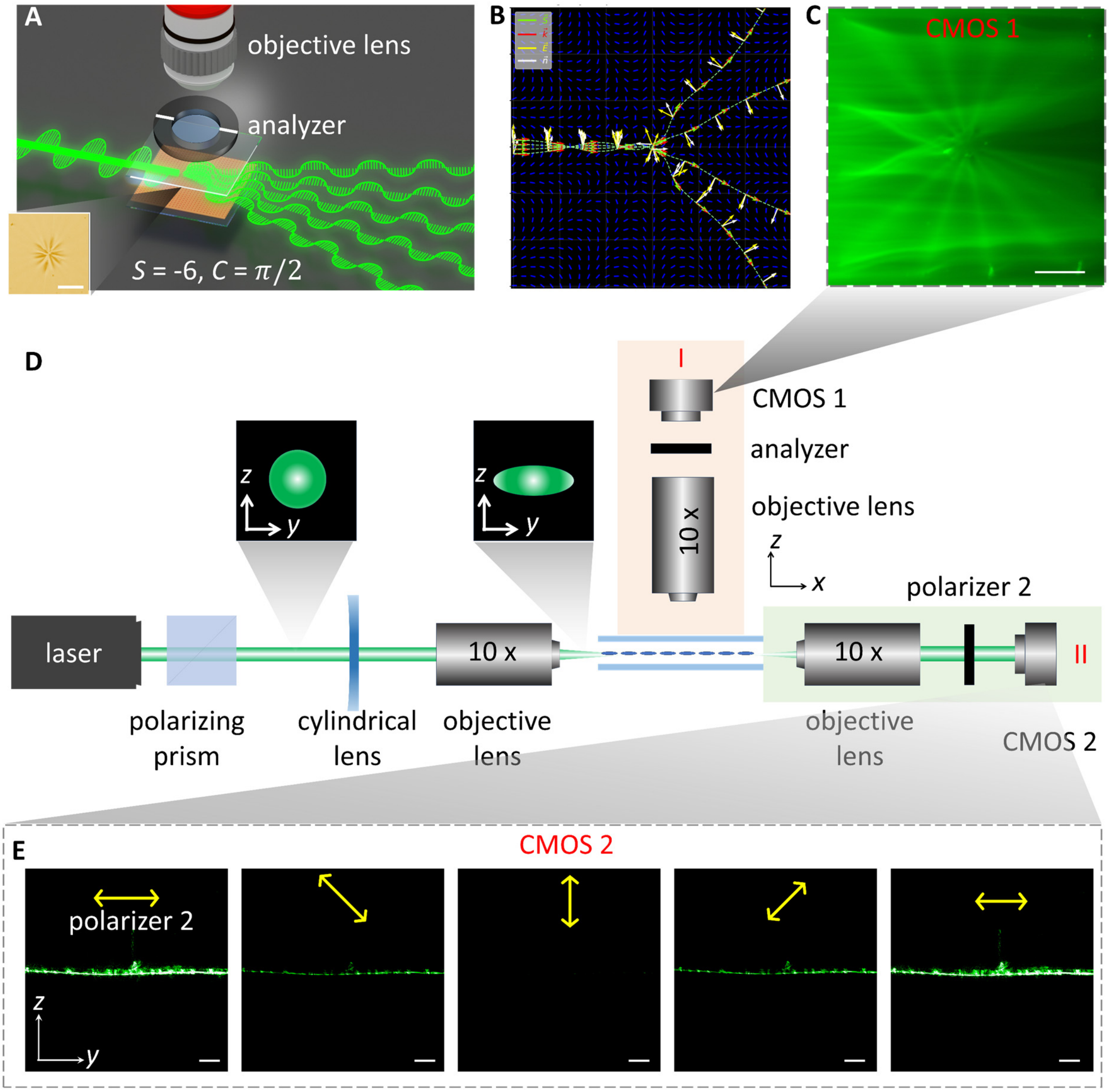

Fig. S8. Simulation and experimental detection of the polarization of in-plane optical waves. (**A**) Schematic illustration of a five-branched optical field induced by a topological vortex with S = -6 and angular offset $C = \pi/2$. The incident beam is linearly polarized along the y-axis and maintains its polarization within the in-plane NLC layer throughout propagation. Inset diagram represents the bright-field image of the defect lines. Scale bar: 100 μm. (**B**) Computational simulation showing the spatial evolution of the optical parameters, including the electric field vector, wave vector, and Poynting vector. The blue rods in the background represent the local director distribution. (**C**) Top-view optical micrographs of the in-plane light field under quasi-plane wave incidence, captured using a polarized optical microscope. Scale bar: 200 μm. (**D**) Schematic of the experimental setup used to investigate the optical incidence and detection (Path II) and visualize the in-plane light trajectories (Path I). (**E**) Experimental characterization of the polarization state of the in-plane beam transformation by analyzing the output signals. Yellow arrows indicate the optical axis orientation of polarizer 2. Scale bar: 1 mm.

Supplementary Figure S9

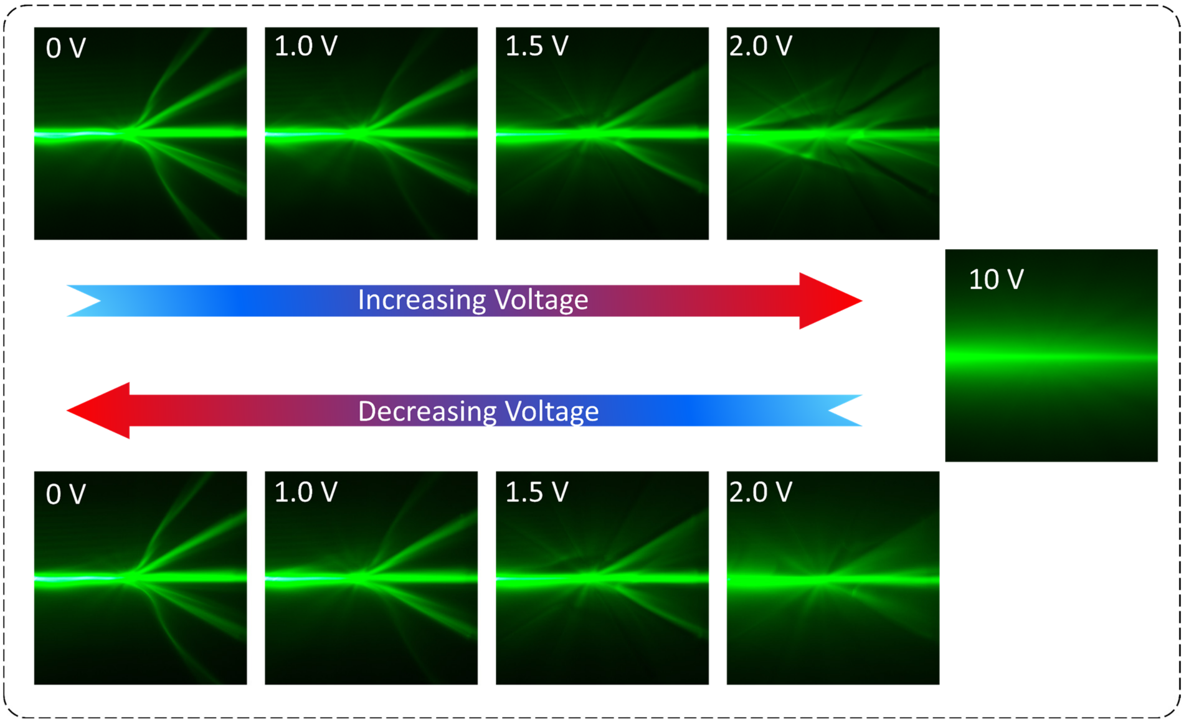

Fig. S9. Reconfigurable optical branching under applied AC electric fields. Optical branching patterns are dynamically modulated by sinusoidal AC electric fields with a constant frequency of f = 1 kHz. The applied voltage is gradually increased and then decreased within the range of 1 V to 10 V, enabling reversible and reconfigurable light propagation behaviors in the NLC medium.

Supplementary Figure S10

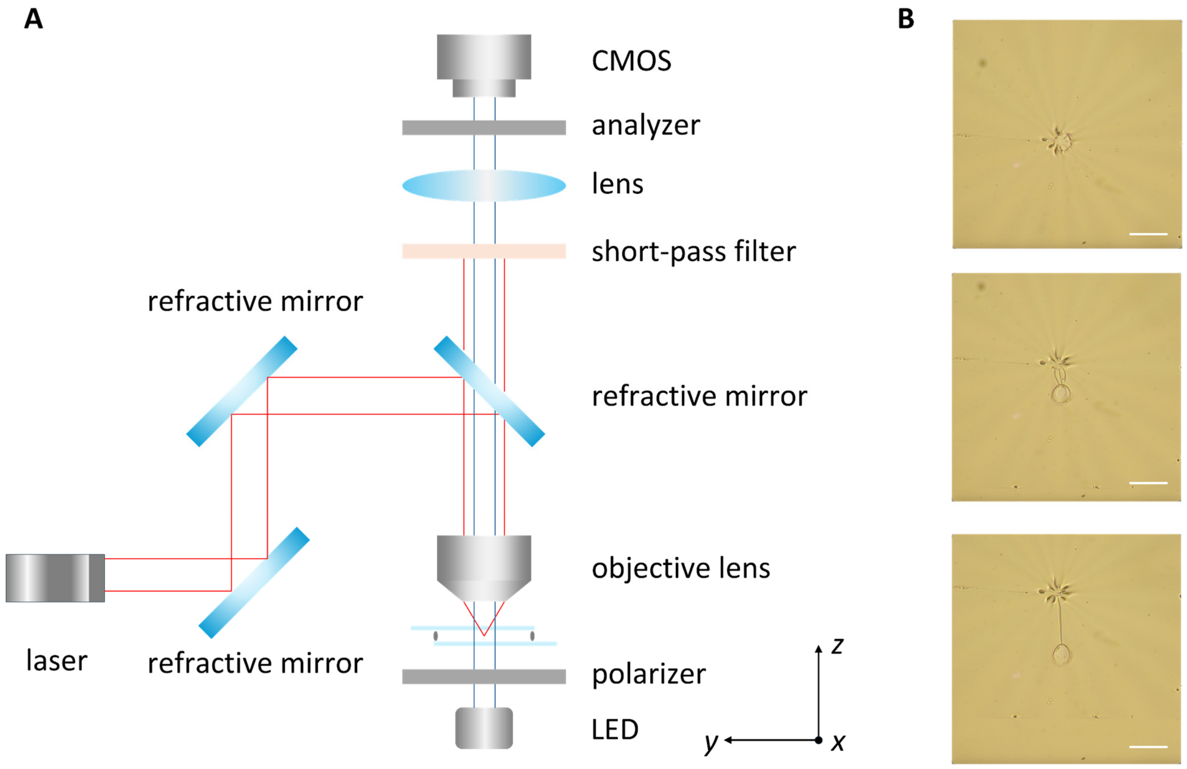

Fig. S10. Experimental setup for polarized optical microscope and laser tweezers. (**A**) Schematic diagram of the optical setup combining polarized optical microscopy and laser tweezers. The optical path of the 1064-nm laser used for optical trapping is indicated by red lines, while the light path of the microscope is shown in blue. A short-pass filter is employed to block the 1064-nm wavelength while allowing shorter wavelengths to pass for imaging purposes. (**B**) Bright-field microscopy images showing the dynamic manipulation of defect lines by activating the laser tweezers. Scale bar: 200 µm.

Description of Movie S1 (separate file)

Electrical tuning of branches number in chaotic optical branching. The branching originates from a vortex core with a deterministic winding number and angular offset (S = -6, C = π/2), with the core marked by an orange arrow. Inset diagrams show the corresponding simulated/experimental POM micrograph (upper left) and director distribution (lower left). An external electric field at f = 1 kHz is swept between 0 V and 10 V. During the voltage increase, the branching pattern evolves from 1×5 to 1×3 and finally 1×1, returning to its initial state during the reverse sweep. Instantaneous voltages are indicated in the upper-right corner of each frame. The scale bar represents 200 μm.

Description of Movie S2 (separate file)

Example showing electrically reconfigurable branching driven by the relaxation of morphed defect lines with *S* = -6, *C* = π/2. Bright-field imaging captures optical branches originating from a vortex with a deterministic winding number and angular offset (S = -6, C = π/2). Under a fixed external field (f = 1 kHz, V = 1.5 V), the defect gradually morphed altering the branching pattern. Over 30 s, the system approaches a steady state and returns to its initial configuration once the field is switched off. Insets show the initial POM texture (upper left) and director field (lower left); a clock trace at the center records the elapsed time.

Description of Movie S3 (separate file)

Example showing electrically reconfigurable branching driven by the relaxation of morphed defect lines with *S* = -4, *C* = π/2. Optical branches originate from a vortex with a deterministic winding number and angular offset (S = -4, C = π/2). Under a fixed external field (f = 1 kHz, V = 2 V), gradual morphing of the defect alters the branching pattern. Within 20 s, the system reaches a steady state and recovers its initial configuration when the field is turned off. Insets show the initial POM texture (upper left) and director field (lower left); a clock trace at the center records the elapsed time.

Description of Movie S4 (separate file)

Laser tweezers–induced stretching of defect and particle capture. Bright-field imaging shows manipulation of a defect core using optical tweezers. The left panel demonstrates the extension of

a deformable defect line (S = -5, $C = \pi/2$) following the tweezer path. The right panel shows a rod-like particle (100 μm in length) being captured by the tweezers and escaping from the vortex core (S = -6, $C = \pi/2$). The laser power is gradually increased to 1 W, inducing a phase transition of the NLC from the nematic to the isotropic state.

Description of Movie S5 (separate file)

Perturbation of chaotic scattering induced by slight variations in the incident position y_0. The movie demonstrates the sensitivity of the chaotic optical branching to initial conditions. A minimal change in the incident beam position y_0 leads to pronounced alterations in the resulting scattering pattern, highlighting the intrinsic chaotic nature of the system. Insets display the POM texture (upper left) and corresponding director landscape (lower left) of the vortex (S = -7, $C = \pi/2$). The incident position is dynamically marked by green arrows in the POM inset, while the core position is indicated by a yellow arrow enclosed by a dashed circle.

Description of Movie S6 (separate file)

Experimental method for determining the sign of the winding number. POM micrographs under crossed polarizers (P and A) show two topological vortices with opposite winding numbers: S = 2 (left panel) and S = -2 (right panel). When the analyzer is rotated anticlockwise, the dark brushes of the S = 2 vortex rotate in the same direction, whereas those of the S = -2 vortex rotate in the opposite (clockwise) direction, providing a straightforward method to determine the sign of the winding number.

REFERENCES

1. S. H. Strogatz, *Nonlinear Dynamics and Chaos: With Applications to Physics, Biology, Chemistry, and Engineering* (CRC, Boca Raton, ed. 2, 2015).

2. E. N. Lorenz, Energy and numerical weather prediction. *Tellus* **12**, 364–373 (2024).

3. T. Shinbrot, C. Grebogi, J. Wisdom, J. A. Yorke, Chaos in a double pendulum. *Am. J. Phys.* **60**, 491–499 (1992).

4. X. Zeng, R. A. Pielke, R. Eykholt, Chaos theory and its applications to the atmosphere. *Bull. Amer. Meteor. Soc.* **74**, 631–644 (1993).

5. J. R. Ackerhalt, P. W. Milonni, M. L. Shih, Chaos in quantum optics. *Phys. Rep.* **128**, 205–300 (1985).

6. A. Argyris, D. Syvridis, L. Larger, V. Annovazzi-Lodi, P. Colet, I. Fischer, J. García-Ojalvo, C. R. Mirasso, L. Pesquera, K. A. Shore, Chaos-based communications at high bit rates using commercial fibre-optic links. *Nature* **438**, 343–346 (2005).

7. F. T. Arecchi, G. Giacomelli, P. L. Ramazza, S. Residori, Experimental evidence of chaotic itinerancy and spatiotemporal chaos in optics. *Phys. Rev. Lett.* **65**, 2531–2534 (1990).

8. O. De Feo, Self-emergence of chaos in the identification of irregular periodic behavior. *Chaos* **13**, 1205–1215 (2003).

9. S. Shinohara, T. Harayama, T. Fukushima, M. Hentschel, S. Sunada, E. E. Narimanov, Chaos-assisted emission from asymmetric resonant cavity microlasers. *Phys. Rev. A* **83**, 053837 (2011).

10. K. J. Vahala, Optical microcavities. *Nature* **424**, 839–846 (2003).

11. N. J. Balmforth, G. R. Ierley, E. A. Spiegel, Chaotic pulse trains. *SIAM J. Appl. Math.* **54**, 1291–1334 (1994).

12. J. M. Dudley, F. Dias, M. Erkintalo, G. Genty, Instabilities, breathers and rogue waves in optics. *Nat. Photonics* **8**, 755–764 (2014).

13. Z. Wang, K. Zhu, F. Zhang, Q. Gong, Q.-F. Yang, Collision-induced rogue waves and universal transport dynamics in chaotic Kerr microresonators. *Sci. Adv.* **11**, eadv9422 (2025).

14. L. Wang, X. Mao, A. Wang, Y. Wang, Z. Gao, S. Li, L. Yan, Scheme of coherent optical chaos communication. *Opt. Lett.* **45**, 4762–4765 (2020).

15. T. Steinle, J. N. Greiner, J. Wrachtrup, H. Giessen, I. Gerhardt, Unbiased all-optical random-number generator. *Phys. Rev. X* **7**, 041050 (2017).

16. S.-S. Chang, K.-H. Wu, S.-J. Liu, Z.-K. Lin, J.-B. Wu, S.-J. Ge, L.-J. Chen, P. Chen, W. Hu, Y. Xu, H. Chen, D. He, D.-Q. Yang, J.-H. Jiang, Y.-Q. Lu, J.-H. Chen, Electrical tuning of branched flow of light. *Nat. Commun.* **15**, 197 (2024).

17. L. Fan, X. Yan, H. Wang, L. V. Wang, Real-time observation and control of optical chaos. *Sci. Adv.* **7**, eabc8448 (2021).

18. S.-Y. Lee, M. S. Kurdoglyan, S. Rim, C.-M. Kim, Resonance patterns in a stadium-shaped microcavity. *Phys. Rev. A* **70**, 023809 (2004).

19. T. Liu, L. Shen, Fluid flow and optical flow. *J. Fluid Mech.* **614**, 253–291 (2008).

20. A. Patsyk, U. Sivan, M. Segev, M. A. Bandres, Observation of branched flow of light. *Nature* **583**, 60–65 (2020).

21. E. J. Heller, R. Fleischmann, T. Kramer, Branched flow. *Phys. Today* **74**, 44–51 (2021).

22. H. Degueldre, J. J. Metzger, T. Geisel, R. Fleischmann, Random focusing of tsunami waves. *Nat. Phys.* **12**, 259–262 (2016).

23. L. H. Ying, Z. Zhuang, E. J. Heller, L. Kaplan, Linear and nonlinear rogue wave statistics in the presence of random currents. *Nonlinearity* **24**, R67–R87 (2011).

24. M. A. Wolfson, S. Tomsovic, On the stability of long-range sound propagation through a structured ocean. *J. Acoust. Soc. Am.* **109**, 2693–2703 (2001).

25. A. Brandstötter, A. Girschik, P. Ambichl, S. Rotter, Shaping the branched flow of light through disordered media. *Proc. Natl. Acad. Sci. U.S.A.* **116**, 13260–13265 (2019).

26. X. Yu, X.-Y. Fang, J.-Q. Tian, X.-Z. Tang, Z.-Y. Wang, J.-H. Chen, Y.-Q. Lu, B.-X. Li, On-demand tailoring of optical branched flow via soft matter domain engineering. *Laser Photonics Rev.* **19**, 2401717 (2025).

27. S.-K. Zhu, Z.-H. Zheng, W. Meng, S.-S. Chang, Y. Tan, L.-J. Chen, X. Fang, M. Gu, J.-h. Chen, Harnessing disordered photonics via multi-task learning towards intelligent four-dimensional light field sensors. *PhotoniX* **4**, 26 (2023).

28. H. S. Yun, D. Wei, S. Yang, G. Park, M. S. Kim, T. J. Shin, D. M. Walba, M. J. Han, D. K. Yoon, Reconfigurable liquid crystal-based physical unclonable function integrating optical and electrical responses. *Adv. Mater.* **37**, e2504288 (2025).

29. C. Meng, J.-S. Wu, Ž. Kos, J. Dunkel, C. Nisoli, I. I. Smalyukh, Emergent dimer-model topological order and quasiparticle excitations in liquid crystals: Combinatorial vortex lattices. *Phys. Rev. X* **15**, 021084 (2025).

30. H. Zhao, B. A. Malomed, I. I. Smalyukh, Topological solitonic macromolecules. *Nat. Commun.* **14**, 4581 (2023).

31. O. D. L. Maurice Kleman, *Soft Matter Physics: An Introduction* (Springer, 2003).

32. M. Kleman, O. D. Lavrentovich, Topological point defects in nematic liquid crystals. *Philos. Mag.* **86**, 4117–4137 (2006).

33. Y. Sasaki, V. S. R. Jampani, C. Tanaka, N. Sakurai, S. Sakane, K. V. Le, F. Araoka, H. Orihara, Large-scale self-organization of reconfigurable topological defect networks in nematic liquid crystals. *Nat. Commun.* **7**, 13238 (2016).

34. I. Dierking, M. Ravnik, E. Lark, J. Healey, G. P. Alexander, J. M. Yeomans, Anisotropy in the annihilation dynamics of umbilic defects in nematic liquid crystals. *Phys. Rev. E* **85**, 021703 (2012).

35. Z. Zhao, H. Li, Y. Yao, Y. Zhao, F. Serra, K. Kawaguchi, H. Zhang, M. Sano, Integer topological defects offer a methodology to quantify and classify active cell monolayers. *Nat. Commun.* **16**, 2452 (2025).

36. R. Zhang, A. Mozaffari, J. J. de Pablo, Autonomous materials systems from active liquid crystals. *Nat. Rev. Mater.* **6**, 437–453 (2021).

37. P. Chen, B.-Y. Wei, W. Hu, Y.-Q. Lu, Liquid-crystal-mediated geometric phase: From transmissive to broadband reflective planar optics. *Adv. Mater.* **32**, e1903665 (2020).

38. Y. Guo, M. Jiang, C. Peng, K. Sun, O. Yaroshchuk, O. Lavrentovich, Q.-H. Wei, High-resolution and high-throughput plasmonic photopatterning of complex molecular orientations in liquid crystals. *Adv. Mater.* **28**, 2353–2358 (2016).

39. J. V. Selinger, *Introduction to the Theory of Soft Matter: From Ideal Gases to Liquid Crystals* (Springer, 2016).

40. S.-T. W. Deng-Ke Yang, *Fundamentals of Liquid Crystal Devices* (Wiley, 2014).

41. X. Yu, S.-S. Chang, Z.-Y. Wang, J. Liu, X.-Z. Tang, J.-H. Chen, B.-X. Li, Y.-Q. Lu, Dynamic transition from branched flow of light to beam steering in disordered nematic liquid crystal. *Laser Photonics Rev.* **18**, 2400366 (2024).

42. C. Meng, J.-S. Wu, I. I. Smalyukh, Topological steering of light by nematic vortices and analogy to cosmic strings. *Nat. Mater.* **22**, 64–72 (2023).

43. R. Wang, B. Ying, S. Shi, J. Wang, B.-Z. Wang, M. Liang, Y. Shen, Hybrid electromagnetic toroidal vortices. *Sci. Adv.* **11**, eads4797 (2025).

44. H. Ge, X.-Y. Xu, L. Liu, R. Xu, Z.-K. Lin, S.-Y. Yu, M. Bao, J.-H. Jiang, M.-H. Lu, Y.-F. Chen, Observation of acoustic skyrmions. *Phys. Rev. Lett.* **127**, 144502 (2021).

45. B. Wang, Z. Che, C. Cheng, C. Tong, L. Shi, Y. Shen, K. Y. Bliokh, J. Zi, Topological water-wave structures manipulating particles. *Nature* **638**, 394–400 (2025).

46. B.-X. Li, V. Borshch, R.-L. Xiao, S. Paladugu, T. Turiv, S. V. Shiyanovskii, O. D. Lavrentovich, Electrically driven three-dimensional solitary waves as director bullets in nematic liquid crystals. *Nat. Commun.* **9**, 2912 (2018).

47. N. P. Haputhanthrige, M. Rajabi, O. D. Lavrentovich, Effects of photopatterning conditions on azimuthal surface anchoring strength. *Crystals* **14**, 1058 (2024).

48. I. I. Smalyukh, S. V. Shiyanovskii, O. D. Lavrentovich, Three-dimensional imaging of orientational order by fluorescence confocal polarizing microscopy. *Chem. Phys. Lett.* **336**, 88–96 (2001).

49. I. I. Smalyukh, Y. Lansac, N. A. Clark, R. P. Trivedi, Three-dimensional structure and multistable optical switching of triple-twisted particle-like excitations in anisotropic fluids. *Nat. Mater.* **9**, 139–145 (2010).

50. M. V. Berry, C. Upstill, "IV Catastrophe optics: Morphologies of caustics and their diffraction patterns," in *Progress in Optics*, E. Wolf, Ed. (Elsevier, 1980), vol. 18, pp. 257–346.

51. J. J. Metzger, R. Fleischmann, T. Geisel, Intensity fluctuations of waves in random media: What is the semiclassical limit? *Phys. Rev. Lett.* **111**, 013901 (2013).

52. M. V. Berry, The singularities of light: Intensity, phase, polarisation. *Light Sci. Appl.* **12**, 238 (2023).

53. M. Peccianti, C. Conti, G. Assanto, A. De Luca, C. Umeton, Routing of anisotropic spatial solitons and modulational instability in liquid crystals. *Nature* **432**, 733–737 (2004).

54. J. J. Sandford O'Neill, P. S. Salter, M. J. Booth, S. J. Elston, S. M. Morris, Electrically-tunable positioning of topological defects in liquid crystals. *Nat. Commun.* **11**, 2203 (2020).

55. J.-S. B. Tai, I. I. Smalyukh, Three-dimensional crystals of adaptive knots. *Science* **365**, 1449–1453 (2019).

56. J.-S. B. Tai, A. J. Hess, J.-S. Wu, I. I. Smalyukh, Field-controlled dynamics of skyrmions and monopoles. *Sci. Adv.* **10**, eadj9373 (2024).

57. C. Letellier, E. M. A. M. Mendes, J.-M. Malasoma, Lorenz-like systems and Lorenz-like attractors: Definition, examples, and equivalences. *Phys. Rev. E* **108**, 044209 (2023).

58. Z.-G. Zheng, Y. Li, H. K. Bisoyi, L. Wang, T. J. Bunning, Q. Li, Three-dimensional control of the helical axis of a chiral nematic liquid crystal by light. *Nature* **531**, 352–356 (2016).

59. A. J. Hess, G. Poy, J.-S. B. Tai, S. Žumer, I. I. Smalyukh, Control of light by topological solitons in soft chiral birefringent media. *Phys. Rev. X* **10**, 031042 (2020).

60. H. Wu, N. Mata-Cervera, H. Wang, Z. Zhu, C. Qiu, Y. Shen, Photonic torons with 3D topology transitions and tunable spin monopoles. *Phys. Rev. Lett.* **135**, 063802 (2025).

61. C. Sheng, H. Liu, H. Chen, S. Zhu, Definite photon deflections of topological defects in metasurfaces and symmetry-breaking phase transitions with material loss. *Nat. Commun.* **9**, 4271 (2018).

62. M. A. Topinka, B. J. LeRoy, R. M. Westervelt, S. E. J. Shaw, R. Fleischmann, E. J. Heller, K. D. Maranowski, A. C. Gossard, Coherent branched flow in a two-dimensional electron gas. *Nature* **410**, 183–186 (2001).

63. Y. Arakawa, S. Nakajima, S. Kang, M. Shigeta, G.-i. Konishi, J. Watanabe, Design of an extremely high birefringence nematic liquid crystal based on a dinaphthyl-diacetylene mesogen. *J. Mater. Chem.* **22**, 13908–13910 (2012).

64. Y.-Q. Lu, Y. Li, Planar liquid crystal polarization optics for near-eye displays. *Light Sci. Appl.* **10**, 122 (2021).

65. K. Yin, E.-L. Hsiang, J. Zou, Y. Li, Z. Yang, Q. Yang, P.-C. Lai, C.-L. Lin, S.-T. Wu, Advanced liquid crystal devices for augmented reality and virtual reality displays: Principles and applications. *Light Sci. Appl.* **11**, 161 (2022).

66. R. Zhang, Z. Zhang, J. Han, L. Yang, J. Li, Z. Song, T. Wang, J. Zhu, Advanced liquid crystal-based switchable optical devices for light protection applications: Principles and strategies. *Light Sci. Appl.* **12**, 11 (2023).

67. D. Wang, Y.-L. Li, F. Chu, N.-N. Li, Z.-S. Li, S.-D. Lee, Z.-Q. Nie, C. Liu, Q.-H. Wang, Color liquid crystal grating based color holographic 3D display system with large viewing angle. *Light Sci. Appl.* **13**, 16 (2024).

68. H. L. Ong, M. Schadt, I. F. Chang, Material parameters and intrinsic optical bistability in room temperature nematics RO-TN-200, -201, -403, E7, m1, and m3. *Mol. Cryst. Liq. Cryst.* **132**, 45–52 (1986).

69. R. Wald, *General Relativity* (University of Chicago Press, 2010).

70. M. Ravnik, S. Žumer, Landau–de Gennes modelling of nematic liquid crystal colloids. *Liq. Cryst.* **36**, 1201–1214 (2009).

71. G. Poy, A. J. Hess, I. I. Smalyukh, S. Žumer, Chirality-enhanced periodic self-focusing of light in soft birefringent media. *Phys. Rev. Lett.* **125**, 077801 (2020).

72. G. Poy, S. Žumer, Ray-based optical visualisation of complex birefringent structures including energy transport. *Soft Matter* **15**, 3659–3670 (2019).

73. M. Nikkhou, M. Škarabot, S. Čopar, M. Ravnik, S. Žumer, I. Muševič, Light-controlled topological charge in a nematic liquid crystal. *Nat. Phys.* **11**, 183–187 (2015).

74. Z.-Y. Wang, Z. Zhou, H. Zhang, Y. Wei, H.-G. Yu, W. Hu, W. Chen, H.-T. Dai, L.-L. Ma, C.-W. Qiu, Y.-Q. Lu, Vectorial liquid-crystal holography. *eLight* **4**, 5 (2024).
